# Diet and Gut Microbiota in Inflammatory Bowel Disease: A Clinical and Nutritional Perspective

**DOI:** 10.3390/ph19020318

**Published:** 2026-02-14

**Authors:** Luisa Bertin, Sonia Facchin, Brigida Barberio, Daria Maniero, Greta Lorenzon, Francesco Cesaroni, Miriana Zanconato, Giulia Romanelli, Francesco Francini-Pesenti, Luca Busetto, Mara Cananzi, Paola Gaio, Luca Bosa, Fabiana Zingone, Laura Gianolio, Oriana M. Damas, Edoardo Vincenzo Savarino

**Affiliations:** 1 Department of Surgery, Oncology, Gastroenterology, University of Padua, 35128 Padova, Italy; luluisa92@gmail.com (L.B.); dariamaniero@gmail.com (D.M.); fabiana.zingone@unipd.it (F.Z.); 2 Gastroenterology Unit, Azienda Ospedale–Università Padova, 35128 Padova, Italy; 3 Dietetics and Clinical Nutrition Unit, Padova University Hospital, 35128 Padova, Italy; 4 Clinical Nutrition Unit, Department of Medicine, University of Padova, 35128 Padova, Italy; 5 Department of Medicine, University of Padova, 35128 Padova, Italy; 6 Unit of Pediatric Gastroenterology, Digestive Endoscopy, Hepatology and Care of the Child with Liver Transplantation, Department of Women’s and Children’s Health, University Hospital of Padova, 35128 Padova, Italy; 7 Department of Paediatrics, Vittore Buzzi Children’s Hospital, University of Milan, 20154 Milan, Italy; 8 Crohn’s and Colitis Center, Division of Digestive Health and Liver Diseases, School of Medicine, University of Miami Miller, Miami, FL 33136, USA; omazorra@med.miami.edu

**Keywords:** microbiome, diet, IBD, CD, UC, CDED, exclusive enteral nutrition, mediterranean diet

## Abstract

Inflammatory bowel diseases, comprising Crohn’s disease and ulcerative colitis, represent chronic inflammatory disorders with rising global incidence, underscoring the pivotal role of modifiable environmental factors in disease pathogenesis. Diet and intestinal microbiota have emerged as critical bidirectional therapeutic targets through complex interactions with host immune responses. Epidemiological evidence demonstrates that healthy and high fiber diets reduce disease risk, while ultra-processed foods and inflammatory dietary patterns increase susceptibility. Therapeutic nutritional interventions, including exclusive enteral nutrition, the Crohn’s Disease Exclusion Diet combined with partial enteral nutrition, and the Mediterranean diet can induce and maintain clinical remission while promoting favorable microbiome modifications characterized by the enrichment of butyrate-producing taxa such as *Faecalibacterium prausnitzii* and *Roseburia* species, alongside a reduction in pathogenic Proteobacteria. Micronutrient deficiencies affect up to 78% of patients through malabsorption, chronic blood losses, dietary restrictions, and drug–nutrient interactions. Nutritional status significantly impacts surgical outcomes, with preoperative malnutrition and sarcopenia associated with increased postoperative complications, and it reciprocally influences biologic therapy response. Integration of personalized, microbiome-informed dietary strategies as complementary components of comprehensive treatment plans represents a promising therapeutic frontier, requiring multidisciplinary collaboration, rigorous clinical trials with standardized microbiome analyses, and precision nutrition algorithms accounting for disease phenotype, baseline microbial composition, and individual patient characteristics to optimize outcomes and improve quality of life.

## 1. Introduction

Inflammatory bowel diseases (IBD), comprising Crohn’s disease (CD) and ulcerative colitis (UC), are chronic inflammatory disorders of the gastrointestinal tract characterized by relapsing and remitting courses that significantly impair quality of life [1,2,3,4,5,6]. These conditions arise from complex interactions between genetic susceptibility, immune dysregulation, and environmental factors, with their rising global incidence, particularly in newly industrialized countries, highlighting the critical role of modifiable environmental determinants in disease pathogenesis [7,8]. Among these factors, diet has emerged as a key player, influencing both disease onset and progression through its profound effects on intestinal inflammation and microbial ecology. The intestinal microbiota serves as a crucial interface between dietary components and host immune responses, with physiological microbial communities contributing to vitamin synthesis, pathogen control, and nutrient metabolism [4,5,9,10,11]. The human gut microbiome is dominated by two main phyla: *Firmicutes* (including butyrate-producing genera such as *Faecalibacterium*, *Roseburia*, *Eubacterium*, and *Ruminococcus*) and *Bacteroidetes* (including *Bacteroides* and *Prevotella*). Short-chain fatty acids (SCFAs), particularly butyrate, produced by beneficial bacteria, are essential for colonocyte nutrition and intestinal barrier integrity. In patients with IBD, characteristic dysbiosis occurs, marked by reduced diversity, depletion of butyrate-producing *Firmicutes* such as *Faecalibacterium prausnitzii* and *Roseburia* species, and expansion of potentially pathogenic *Proteobacteria*, including *Escherichia coli* and *Klebsiella* species [10,12]. This altered microbial composition compromises SCFA production, weakens intestinal barrier integrity, and perpetuates inflammatory cascades that drive disease activity [13]. A recent systematic review with an integrated bioinformatic reanalysis of 18 treatment-naïve cohorts (2160 samples from 1743 individuals with new-onset IBD) provides the most comprehensive characterization of microbial perturbations at disease onset [14]. Using a unified QIIME2 pipeline with multivariable modeling to control for methodological heterogeneity across studies, this analysis demonstrated that the core dysbiosis signature in both CD and UC comprises broad depletion of obligate anaerobic bacteria alongside enrichment of oxygen-tolerant organisms, including aerobic (*Pseudomonas*, *Schaalia*), microaerophilic (*Campylobacter*, *Dialister*), and facultative anaerobic genera (*Haemophilus*, *Enterococcus*, *Rothia*). Depletion of *Alistipes*, *Roseburia*, and *Phascolarctobacterium* was consistently observed across both CD and UC compared to controls. Notably, enrichment of oral cavity-associated genera, including *Fusobacterium*, *Peptostreptococcus*, *Haemophilus*, *Veillonella*, and *Granulicatella*, suggests a pathogenic role for oral–gut bacterial transmission in IBD pathogenesis. These findings support the “oxygen hypothesis” of IBD dysbiosis and highlight luminal oxygen modulation as a potential therapeutic target at disease onset.

Epidemiological evidence demonstrates that specific dietary patterns substantially modify IBD risk, with healthy diets conferring protection, while inflammatory diets rich in ultra-processed foods increase disease susceptibility [15,16]. Nutritional interventions have emerged as promising therapeutic strategies, with exclusive enteral nutrition (EEN) achieving remission rates comparable to corticosteroids in pediatric CD, and solid food-based approaches, including the Crohn’s Disease Exclusion Diet (CDED) combined with partial enteral nutrition (PEN), demonstrating the capacity to induce clinical remission while promoting favorable microbiome modifications [17]. However, translating these findings into clinical practice remains challenging, as the evidence base is characterized by heterogeneous study designs, inconsistent outcome measures, and, for many dietary approaches, conflicting results between observational and interventional data. Notably, dietary associations with IBD risk and disease course frequently differ between CD and UC, underscoring the need for disease-specific dietary recommendations.

The therapeutic effects of dietary interventions in IBD are increasingly understood to be mediated by microbiota-derived bioactive metabolites, including short-chain fatty acids, bile acids, tryptophan derivatives, and antimicrobial peptides, which collectively modulate intestinal barrier integrity, immune regulation, and pathogen resistance [18,19]. This metabolite-centric perspective underscores the importance of understanding not only microbial community composition but also the functional metabolic output of the gut microbiome in response to dietary modification.

Beyond macronutrient composition, micronutrient deficiencies represent frequent complications in IBD, affecting up to 78% of patients through mechanisms encompassing malabsorption, chronic blood losses, dietary restrictions, and drug–nutrient interactions, with iron, zinc, calcium, vitamin D, vitamin B12, and folate being particularly vulnerable [20,21,22,23,24,25,26]. Pharmacological therapies commonly employed in IBD management significantly impact nutritional status [27,28,29,30]. Emerging therapeutic strategies, including fecal microbiota transplantation (FMT) and probiotic engineering, offer novel approaches to directly modulate intestinal microbial communities, as clearly demonstrated in *Clostridioides difficile* infection, though optimal patient selection, standardization of preparation and delivery protocols, and long-term efficacy remain areas of active investigation [31,32,33,34,35]. Importantly, bacterial strains rather than species may be more strongly associated with IBD and more reproducible across cohorts as biomarkers. Strain-level analysis has revealed that disease-associated strains demonstrate competitive dominance over their healthy counterparts, and the absence of health-associated strains, such as specific *Eggerthella lenta* strains, correlates with elevated fecal calprotectin levels [36]. This finding has implications for both diagnostic biomarker development and therapeutic targeting, suggesting that species-level analyses may overlook clinically relevant functional heterogeneity.

This comprehensive review examines the multifaceted relationships between diet, intestinal microbiota, and IBD management, analyzing dietary risk factors for disease incidence and progression, effects of specific nutritional interventions on microbiota composition and clinical outcomes, micronutrient roles and deficiency implications, drug–nutrient interactions, and emerging microbiome-targeted therapies. The objective is to provide an evidence-based synthesis of current knowledge to guide the integration of nutritional and microbiome-informed approaches into comprehensive therapeutic strategies for IBD patients, with particular emphasis on intervention personalization based on disease phenotype, baseline microbial status, and individual patient characteristics and preferences to optimize therapeutic outcomes and improve quality of life.

## 2. Dietary Risk Factors for Incident IBD

Early life dietary exposure during the period of maximum vulnerability in the microbiome and developing immune system plays a critical role in modifying IBD risk. In a systematic review of population-based studies, predominantly case–control in design, a history of breastfeeding in infancy was associated with a lower risk of developing CD (OR 0.71, 95% confidence interval ((CI) 0.59–0.85) and UC odds ratio ((OR 0.78), 95% CI 0.67–0.91) [15]. However, recent prospective cohort data challenge this association. Analysis of British birth cohorts with follow-up to age 33–43 years found no association between breastfeeding and IBD risk [37]. Similarly, three Scandinavian prospective birth cohorts from Norway, Sweden, and Denmark found no difference in CD or UC development with any duration of breastfeeding [38], and a Scottish population-based study (1981–2017) showed no difference between exclusive breastfeeding and formula feeding at 6 weeks [39]. These conflicting findings led the 2025 ECCO Consensus to conclude that, while breastfeeding has well-recognized health benefits, current evidence is insufficient to support it as a significant protector against IBD development [40]. The discrepancy may reflect methodological differences, as meta-analyses favoring protection relied heavily on case–control studies prone to recall bias, whereas prospective cohorts showed null associations. An elegant study examining diet at one year of age in Swedish and Norwegian birth cohorts found that children with high diet quality had a reduced risk of IBD compared to those with low diet quality (HR 0.75, 95% CI 0.56–1.00) [41]. Specific food constituents associated with this protective effect included fish and vegetable intake, while consumption of sugar-sweetened beverages was associated with higher IBD risk.

Regarding macronutrients, prospective cohort data from 170,805 women demonstrated that higher intake of long-chain n-3 polyunsaturated fatty acids (PUFAs) comprising docosapentaenoic acid, eicosapentaenoic acid, and docosahexaenoic acid (HR 0.72, 95% CI 0.51–1.01), or a lower n-3/n-6 PUFA ratio, was associated with reduced risk of UC [42]. Conversely, high intake of arachidonic acid, an n-6 PUFA, and the concentration of arachidonic acid in gluteal fat pad biopsies were associated with increased risk of UC (rate ratio 4.16, 95% CI 1.56–11.04) [43]. This association may be modified by host genotype, particularly polymorphisms at the CYP4F3 and FADS2 loci [44].

Initial evidence from both case–control studies and prospective cohorts suggested an inverse association between dietary fiber and risk of IBD. In the Nurses’ Health Study cohorts, women in the highest quintile of dietary fiber intake (median 24 g/day) had a lower risk of developing CD than those in the lowest quintile (HR 0.59, 95% CI 0.39–0.90) [45]. However, the impact of fiber on IBD risk appears to differ by fiber subtype. In the same cohorts, the inverse association with CD was stronger for fiber intake from fruits, while fiber from whole grains, legumes, or cereal was not associated with modified risk [45]. Different sources and subtypes of fiber may differ by solubility, fermentation potential, and content of other potentially bioactive constituents such as polyphenols.

A systematic review and meta-analysis of 72 prospective studies (65 adult cohorts with 2,043,601 participants, mean follow-up 12.8 years, 1902 CD cases, 4617 UC cases) found that inflammatory diet, characterized by high consumption of red and processed meats, refined grains, sugar-sweetened beverages, and low intake of vegetables, fruits, and whole grains (pooled adjusted Hazard Ratio (aHR 1.63)) and ultra-processed foods (pooled aHR 1.71) increased CD risk, while high fiber (pooled aHR 0.53), mediterranean diet (MD) (pooled aHR 0.59), healthy diet (pooled aHR 0.70), and unprocessed/minimally processed foods (pooled aHR 0.71) decreased CD risk; no consistent associations were found between individual foods or dietary patterns and UC risk [16]. A prospective European cohort study of 413,593 participants from eight countries with mean 16.8-year follow-up identified 177 CD and 418 UC cases, finding no association between total protein, animal protein, or vegetable protein intake and IBD risk, but total meat and red meat consumption were associated with UC risk (HR 4th vs. 1st quartile for total meat 1.40, 95% CI 0.99–1.98, p-trend = 0.01; for red meat 1.61, 95% CI 1.10–2.36, p-trend = 0.007), with no associations found between other animal protein sources (processed meat, fish, shellfish, eggs, poultry) and UC, or between any animal protein sources and CD [46].

Recent epidemiological investigations have highlighted the relationship between plant-focused eating patterns and IBD outcomes through two major population studies. The UK Biobank analysis, encompassing 187,888 participants, alongside the EPIC multicenter investigation involving 341,539 individuals across eight European nations, revealed that superior adherence to nutritionally sound plant-centric dietary indices correlated with markedly diminished IBD risk (HR: 0.75 and 0.71, respectively) [47]. Conversely, the critical distinction emerges in dietary quality: consumption patterns dominated by refined carbohydrates, sweetened beverages, and processed sugars demonstrated elevated disease risk (HR: 1.48 and 1.54). Surgical intervention requirements also differed substantially among patients with established IBD (CD and UC combined): wholesome plant-based approaches reduced surgical risk by half (HR: 0.50, P for trend < 0.001), whereas nutritionally poor variants more than doubled surgical necessity (HR: 2.12, P for trend = 0.003). These associations were observed for IBD overall, with subgroup analyses showing similar trends for both CD and UC for disease incidence, though surgery-specific data were not reported separately by IBD subtype.

Dietary micronutrients may also play a role in IBD pathogenesis. An increase in dietary zinc intake was associated with reduced risk of IBD within the Nurses’ Health Study cohorts, an association mechanistically supported by the beneficial impact of zinc on epithelial barrier function in experimental models [48]. With respect to antioxidants, flavones and resveratrol in the diet were associated with reduced risk of CD in a prospective analysis [49].

Food additives may contribute to IBD pathogenesis. In the Nurses’ Health Study cohorts, participants in the highest quartile of ultraprocessed food (UPF) consumption had an increased risk of CD compared to those with low UPF consumption (HR 1.70, 95% CI 1.23–2.35). Specifically, ultraprocessed breads and breakfast foods, sauces, cheeses, spreads, and gravies showed the strongest association with CD risk. Notably, an association with UC risk was not found [50].

Since food is consumed within the context of other dietary items, dietary patterns may be more relevant than individual food groups in informing strategies for IBD prevention and treatment. In a prospective cohort of 83,147 Swedish adults, adherence to an MD pattern was associated with a lower risk of CD (HR 0.42, 95% CI 0.22–0.80) but not UC [51]. An empiric inflammatory dietary potential (EDIP) score derived from the association of foods with inflammatory cytokines in serum was associated with 51% higher risk of CD (HR 1.51, 95% CI 1.10–2.08) in two prospective cohorts [52]. Interestingly, participants who moved from the lowest EDIP tertile at baseline to the highest tertile eight years later experienced a two-fold increase in relative risk of CD (HR 2.05, 95% CI 1.10–3.79) [52].

An emerging area of investigation concerns the role of the gut phageome (viral component of the microbiome) as a mediator of diet–microbiome interactions in CD. A recent multiomics study by Su et al. investigated the relationship between dietary components, gut phageome, bacteriome, and CD risk in 140 subjects (70 CD patients, 70 healthy controls), with epidemiological validation in the UK Biobank cohort (190,293 participants) [53]. Dietary whey protein consumption was associated with reduced CD risk (HR 0.79, 95% CI 0.65–0.99, *p* = 0.036) and showed the largest effect size on gut phageome composition (R^2^ = 0.075). Whey protein consumers exhibited increased phageome alpha diversity and enrichment of beneficial bacteria, including *Streptococcus thermophilus*, *Faecalibacterium*, *Lachnoclostridium*, and *Lactobacillus*, alongside phages targeting pathogenic bacteria. Mechanistically, whey protein induced the release of a novel *Akkermansia* phage (AkkZT003P) that lysed the mucin-foraging bacterium *Akkermansia muciniphila*, thereby unleashing the growth of the probiotic *S. thermophilus*, which exerted anti-inflammatory effects. In murine models (DSS and TNBS colitis), whey protein supplementation attenuated intestinal inflammation, reduced pro-inflammatory cytokines (tumor necrosis factor-alpha (TNF-α), interleukin-1 (IL-1β), IL-6), and increased Muc-2 expression with preserved mucus layer integrity. This study represents the first demonstration of dietary modulation of phage–bacterium–host interactions in CD protection, suggesting that therapeutic strategies incorporating whey protein, targeted phages, and probiotics warrant further exploration.

Beyond dietary factors, physical activity represents another modifiable lifestyle determinant of IBD risk. A prospective UK Biobank cohort study of 355,021 participants followed for 13.6 years identified 2242 incident IBD cases and demonstrated that moderate physical activity was associated with reduced IBD risk (HR 0.80, 95% CI 0.72–0.89) compared to low physical activity, with a nonlinear U-shaped relationship suggesting approximately 2000 MET min/week as optimal [54]. Sedentary behavior independently increased IBD risk (HR 1.03 per additional hour/day, 95% CI 1.01–1.05), with the combination of low physical activity and prolonged sedentary behavior (≥6 h/day) conferring the highest risk. Potential mechanisms underlying these associations include physical activity-induced modifications of gut microbiome composition and improvements in intestinal barrier permeability.

## 3. Diet and Disease Course

The question of an “optimal” IBD diet remains elusive, as emerging evidence suggests that therapeutic efficacy depends critically on baseline microbiome composition and individual patient characteristics. Recent proposals advocate for dysbiosis assessment prior to dietary prescription, enabling personalized therapeutic strategies rather than universal dietary recommendations [55,56]. This approach recognizes that the gut microbiota functions as a metabolic organ, synthesizing vitamins, suppressing pathogenic expansion, and transforming dietary substrates into bioactive compounds. Consequently, characterizing an individual’s microbial community could direct clinicians toward interventions most likely to restore eubiosis and achieve clinical benefit.

Fewer studies have examined dietary risk factors for disease relapse. Regarding red meat intake and flares, the PREDiCCt (Prognostic Effect of Environmental Factors in Crohn’s and Colitis) study, following 520 Patients with UC, found that those with the highest reported meat intake had a two-fold increase in risk of flares compared to those in the lowest category [57]. The ECCO 2025 Consensus indicates that a reduction in red meat can be considered for the maintenance of remission in UC, though there is insufficient evidence to recommend red meat reduction for the induction of remission in either Crohn’s disease or UC, or for the maintenance of remission in Crohn’s disease [40].

In contrast, an Internet-based trial in the IBD Partners cohort found that consumption of two or more servings of red meat per week was not associated with increased risk of disease flares [58], suggesting the need for more rigorously designed studies.

Data on dietary fiber and disease relapse are similarly inconsistent. Two studies showed an inverse association between fiber intake and risk of IBD relapse, while two others found no benefit, and one demonstrated an increased risk of flares with high fiber intake [59,60]. Several potential explanations exist for these divergent findings. Observational studies of fiber and relapse may be subject to reverse causation, as regular flares may alter dietary intake. Differences in fiber source and subtype may also contribute, given variations in solubility, fermentation potential, and other bioactive constituents [61]. Additionally, an individual’s microbiome may influence their ability to process fiber and generate biologically active metabolites, leading to biology-dependent heterogeneity in effect [62]. Studies have highlighted that the generation of beneficial SCFAs from fermentable fiber may be based on inflammatory status, leading to differing effects on the microbiome and metabolome depending on the presence of active disease [63].

Beyond disease activity, diet may influence other outcomes. Low fruit intake was associated with increased occurrence of pouchitis in one study. Mechanistically, fruit consumption was associated with a favorable microbial profile, influencing the abundance of *Faecalibacterium*, *Lachnospira*, and *Ruminococcus* [64].

In line with this, a recent systematic review of 80 controlled clinical trials spanning 13 dietary interventions in healthy and clinical adult populations found that Mediterranean, Japanese, Korean, calorie-restricted, high-polyphenol, high-fiber, plant-based, low-fat, and low-protein diets were associated with increased abundances of short-chain fatty acid- or lactic acid-producing bacteria and reduced opportunistic pathogens, whereas Western, animal-based, low-FODMAP, ketogenic, and gluten-free diets were associated with reduced SCFA-producing bacteria [65]. Notably, while these dietary interventions induced consistent changes at the taxonomic level and in inflammatory biomarkers, they were not consistently associated with shifts in alpha or beta diversity, and substantial methodological heterogeneity across studies was reported [65].

**Figure 1:**
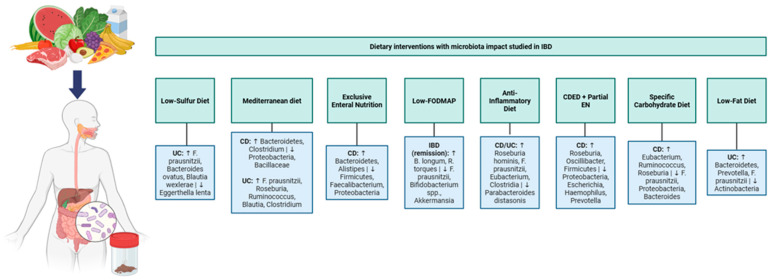
Main microbiome changes occurring during dietary interventions in patients with Inflammatory Bowel Disease. Created in BioRender. Bertin, L. (2026) https://BioRender.com/lz4i4y8. Abbreviations: UC: Ulcerative Colitis; CD: Crohn’s Disease; IBD: Inflammatory Bowel Disease; FODMAP: Fermentable Oligosaccharides, Disaccharides, Monosaccharides, and Polyols; CDED: Crohn’s Disease Exclusion Diet; EN: Enteral Nutrition. ↑ indicates increased relative abundance and ↓ means decreased relative abundance.

The therapeutic potential of dietary interventions lies, at least in part, in their capacity to reshape intestinal microbiome composition and function, although other mechanisms, including direct immunomodulatory effects and gut barrier restoration, likely contribute. Each dietary pattern exerts distinct effects on microbial ecology: some promote butyrate-producing taxa while others reduce pathogenic *Proteobacteria*; some enhance microbial diversity while others restrict fermentable substrates that may exacerbate symptoms in certain patients. Current evidence is summarized in Figure 1. Understanding these microbiome-mediated mechanisms is essential for rational dietary prescription in IBD management.

**Figure 2:**
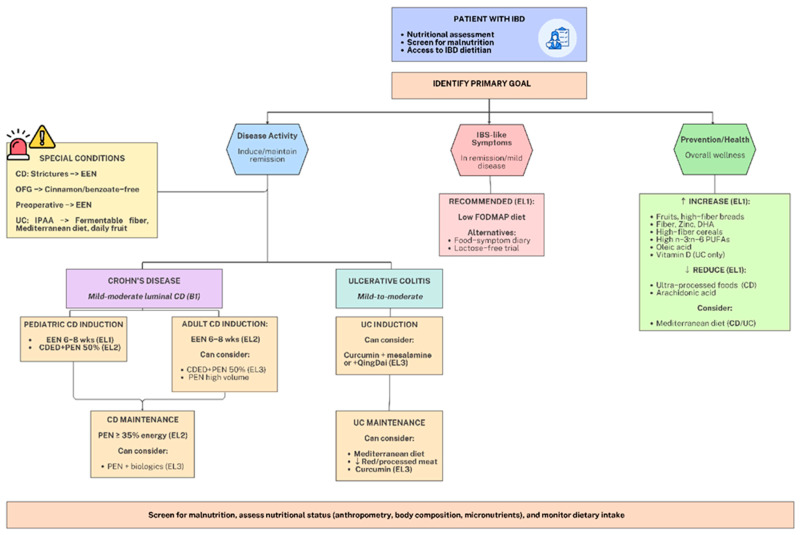
Algorithm for nutritional monitoring and dietary interventions in patients with inflammatory bowel disease, adapted from current clinical practice guidelines [40]. Abbreviations: CD, Crohn’s disease; CDED, Crohn’s Disease Exclusion Diet; DHA, docosahexaenoic acid; EEN, exclusive enteral nutrition; ELI, evidence level indicator; FODMAP, fermentable oligosaccharides, disaccharides, monosaccharides and polyols; IBD, inflammatory bowel disease; IBS, irritable bowel syndrome; IPAA, ileal pouch-anal anastomosis; OFG, orofacial granulomatosis; PEN, partial enteral nutrition; PUFAs, polyunsaturated fatty acids; UC, ulcerative colitis. Within the Prevention/Health and UC maintenance panels, ↑ denotes recommended increases and ↓ denotes recommended reductions in dietary intake.

The ECCO 2025 Consensus provides an evidence-based framework for nutritional monitoring and dietary interventions, synthesizing current recommendations across different disease states and patient populations (Figure 2, Table 1) [40]. The following sections examine major dietary approaches, their mechanisms of action, clinical efficacy, and differential effects on gut microbial communities, providing clinicians with the knowledge necessary to integrate these interventions into comprehensive IBD management strategies.

## 4. Diets for Induction and Maintenance of Remission in Crohn’s Disease

### 4.1. Exclusive Enteral Nutrition

EEN stands as a dietary intervention extensively studied in IBD management, wherein patients receive a nutritionally complete liquid formula as their sole nutritional source for six to eight weeks, with all solid foods eliminated. Water is permitted, and some protocols allow other clear fluids. While this approach demonstrates therapeutic benefit in CD, evidence for UC remains insufficient to support routine recommendation [66].

#### 4.1.1. Clinical Effectiveness

Clinical effectiveness varies considerably by age group. A Cochrane synthesis of ten randomized controlled trials (RCTs) revealed superior outcomes in children compared with adults when comparing EEN against corticosteroids [66]. Intention-to-treat analyses in two pediatric studies showed equivalent remission rates between the two interventions. However, per-protocol analysis excluding children unable to complete therapy due to formula taste aversion or nasogastric tube intolerance demonstrated EEN’s clear advantage, achieving 89% remission versus 61% with corticosteroids [66]. This efficacy profile, combined with avoiding early-life steroid exposure, establishes EEN as preferred initial therapy for pediatric CD [67].

The challenge of treatment adherence remains substantial in both research and clinical settings, primarily stemming from palatability issues, volume consumption difficulties, and tube feeding intolerance [68].

#### 4.1.2. Mechanism of Action and Microbiome Effects

Microbiome alterations during EEN include decreased diversity with reductions in *Firmicutes*, particularly *Faecalibacterium*, *Proteobacteria*, and a decreased *Bacteroides*/*Prevotella* ratio, alongside increased Bacteroidetes, particularly *Alistipes* [69]. Diederen observed elevated *Faecalibacterium* and *Roseburia* abundance among responders [70]. Levine demonstrated that initial microbiome shifts reverted to baseline following habitual diet reintroduction, whereas sustained changes occurred with the CD Exclusion Diet (CDED), including decreased *Haemophilus*, *Veillonella*, *Anaerostipes*, and *Prevotella*, with increased *Roseburia* and *Oscillibacter* [17].

SCFA concentrations decline during EEN but normalize upon solid food reintroduction, accompanied by *Firmicutes* expansion [69,71,72], suggesting that these metabolites may not mediate EEN-induced remission. Conversely, solid exclusion diets demonstrate increased SCFAs and associated bacteria with variable effectiveness [69]. Primary bile acid concentrations decreased after six weeks of EEN, returning to baseline with regular diet resumption, while secondary bile acids increased, approaching healthy control levels. Regarding tryptophan metabolism during EEN and CDED+PEN, decreased kynurenine and quinolinic acid, alongside elevated serotonin metabolites, particularly melatonin and N-acetylserotonin, were observed and strongly associated with sustained remission.

Treatment personalization would require the identification of specific biomarkers that correlate with response to EEN. Employing 16S rRNA V4 sequencing, generating approximately 49,238 reads per sample across 238 operational taxonomic units, investigators found that responders exhibited significantly elevated baseline fecal microbiota Chao1 richness (315 versus 243, *p* = 0.015) despite comparable beta diversity, with 13 differentially abundant taxa [68]. Paradoxically, responders demonstrated substantially reduced pretreatment fecal SCFA concentrations, quantified through gas chromatography and proton nuclear magnetic resonance spectroscopy. Random forest modeling utilizing fecal microbiota composition achieved 79% accuracy with 73% sensitivity and 84% specificity, identifying influential operational taxonomic units assigned to *Bacteroides*, *Lachnospiraceae*, *Ruminococcaceae*, and *Anaerococcus*, all exhibiting higher baseline abundance in non-responders [68]. An integrated multicomponent model incorporating microbiota, metabolites, dietary intake, and clinical parameters achieved 78% accuracy, with an elevated abundance of taxa from *Ruminococcaceae*, *Lachnospiraceae*, and *Bacteroides* alongside phenylacetate, butyrate, and acetate serving as primary predictors of therapeutic failure. Moreover, a prospective investigation of 83 children utilizing Olink proteomics to quantify 92 inflammation-related plasma proteins revealed that CD patients exhibited 29 differentially expressed proteins (26 elevated, 3 decreased) compared with controls, characterized by increased interferon-gamma and Th1-associated proteins, while UC showed Th17 pathway enrichment [73]. Eight weeks of EEN modified 19 proteins (13 increased, including fibroblast growth factor-19, CXCL10, interleukin-10; 6 decreased, including CCL23, interleukin-24, interleukin-6, matrix metalloproteinase-1) with more pronounced effects in patients with ileal involvement and those achieving at least 50% fecal calprotectin (FC) reduction, and machine-learning models using baseline protein profiles predicted EEN response with 89% sensitivity, with thymic stromal lymphopoietin being the most important predictor [73]. Recent mechanistic work has provided clearer insights into how EEN achieves therapeutic benefit. Häcker and colleagues demonstrated that the efficacy of EEN in inducing remission in pediatric CD is mediated through explicit functional alterations of the gut microbiome, despite inherent temporal and individual variability in microbial profiles [74]. This finding suggests that EEN reconstitutes the gut microbiota through functional rather than purely compositional mechanisms, reconciling the apparent paradox of reduced microbial diversity accompanying clinical improvement.

Several unresolved paradoxes characterize EEN therapy. The induced microbial alterations, particularly reduced diversity, contradict prevailing concepts of healthy microbiome composition. A further paradox was described by Gerasimidis et al., who observed that presumptively protective gut bacterial species, including *Faecalibacterium* and other SCFA producers, declined during EEN, yet this reduction was paradoxically associated with disease improvement [75]. This observation suggests that the therapeutic mechanism may operate through reducing exposure to pro-inflammatory dietary components rather than through augmenting beneficial bacteria. Compounding these paradoxes, EEN demonstrates a characteristic rebound phenomenon upon treatment cessation. There is a rapid return of inflammation upon food reintroduction, limiting EEN’s utility as a maintenance strategy [76]. However, recent evidence suggests that the rebound limitation of EEN may be overcome through a cyclical administration strategy. The CD-HOPE trial was an open-label, endpoint-blinded RCT conducted across 21 French hospitals that randomized 100 pediatric patients (median age 12–13 years) with CD in clinical remission (wPCDAI of 12.5 or less) after 6–12 weeks of EEN induction to either cyclic EEN (C-EEN; 100% of daily caloric requirements for 2 weeks every 8 weeks, for at least six cycles) or daily PEN (25% of daily caloric requirements) for 52 weeks [77]. Crucially, both arms used MODULEN IBD, and no concomitant CD medications were administered, making this a trial of drug-free nutritional maintenance. At 12 months, 49% of patients relapsed on C-EEN compared with 76% on PEN (adjusted OR 0.29, 95% CI 0.13–0.70, *p* = 0.0051). These results indicate that reintroducing short courses of complete enteral nutrition at regular intervals is substantially more effective than continuous low-dose supplementation for preventing relapse, and that EEN used cyclically can be reconsidered as a viable maintenance strategy despite the known rebound phenomenon observed with single courses of EEN [77].

Similarly, the initial microbiome shifts revert to baseline following habitual diet reintroduction, and SCFA and primary bile acid concentrations that decreased during EEN return to pre-treatment levels with regular diet resumption [75,78]. This transient nature of EEN-induced changes contrasts with the more durable modifications observed with CDED + PEN, wherein microbial shifts persist throughout the observation period in diet-following patients, providing a rationale for combining dietary approaches with partial enteral nutrition for sustained remission [17]. Additionally, EEN formulations contain substantial additive concentrations, contrasting with therapeutic dietary approaches emphasizing additive and emulsifier minimization [79]. Interpretation of microbiome alterations during EEN must account for substantial methodological heterogeneity across existing trials. A recent review highlighted that EEN studies frequently suffer from small sample sizes, heterogeneous patient populations, inconsistent adherence definitions, and reliance on symptom-based outcomes without objective inflammatory markers [80]. Notably, when fecal gluten immunogenic peptides were employed as an objective adherence marker in children receiving gluten-free EEN, up to 23% of participants had consumed gluten from outside the formula, suggesting that self-reported adherence may overestimate true protocol compliance and complicate interpretation of mechanistic findings [80]. These limitations underscore the need for a standardized trial design incorporating both subjective and objective adherence measures alongside comprehensive microbiome analyses.

Moreover, the exact mechanism of action of EEN remains incompletely understood, particularly given the apparent paradoxes compared to other dietary therapies (reduced microbial diversity despite clinical benefit, high additive content despite emphasis on additive avoidance in other effective diets). Further investigation is required to determine whether biological effects vary by additive type and to elucidate the relative contributions of microbiome modulation, direct anti-inflammatory effects, and gut barrier restoration. 

### 4.2. Partial Enteral Nutrition and CDED

Enteral nutrition strategies in IBD have evolved beyond the exclusive formula approach. When enteral formulas supply approximately 35–50% of daily energy needs alongside conventional food intake, this approach is termed Partial Enteral Nutrition (PEN), offering a practical middle ground between therapeutic effectiveness and patient compliance [81]. Current ECCO guidelines support its use for sustaining remission in CD [40]. The combination of PEN with biological agents, particularly adalimumab, appears to enhance remission induction beyond what monotherapy achieves [82].

A therapeutic diet specifically engineered to mirror the beneficial effects of formula-only nutrition while incorporating solid foods is the CDED, which removes dairy, gluten-containing products, processed meats, animal-derived fats, preserved foods, emulsifiers, and various additives from the diet [83]. This elimination strategy derives from laboratory and animal research suggesting that these components adversely affect intestinal microbial communities and barrier integrity. According to the 2025 ECCO recommendations, combining this exclusion diet with PEN merits consideration for achieving remission in both pediatric and adult CD populations [40].

#### 4.2.1. Clinical Effectiveness

Levine’s pivotal pediatric study randomized 74 children with active CD to receive either the CDED with PEN or formula alone [17]. Treatment tolerance proved superior in the combined approach group at six weeks (97.5% versus 73.7%, *p* = 0.002), while clinical response and remission rates remained comparable between groups initially. However, sustained remission at twelve weeks favored the combined diet approach (75.6% versus 45.1%, *p* = 0.01) as control participants transitioned back to unrestricted eating; among CDED + PEN responders, 87.5% maintained remission through week 12. The dietary intervention similarly reduced inflammatory markers and modified fecal microbial composition as effectively as exclusive formula therapy. In adult populations, Yanai’s comparative study of 44 patients found no significant remission rate differences at six weeks between CDED plus PEN (68.4%) and CDED alone (57.1%, *p* = 0.46). Among the 25 patients achieving remission at week 6, 80% maintained sustained clinical remission through 24 weeks, with 35% achieving endoscopic remission (SES-CD ≤ 3) [84].

Multiple subsequent observational studies have generally confirmed these efficacy findings [85,86]. The international DIETOMICS investigation extended evaluation to a 24-week three-phase CDED protocol—phase 1 (weeks 3–8) with 50% PEN, phase 2 (weeks 9–14) with 25% PEN, and phase 3 (weeks 15–24) with gradual food liberalization—across varying disease severity levels in pediatric populations [87]. Although recruitment challenges during the COVID-19 pandemic limited statistical power, results revealed marked efficacy variation by baseline severity, with 92% achieving remission in mild disease compared to only 25% in severe presentations. Despite six months of dietary restrictions, patients demonstrated significant body mass index Z-score improvements, and only 56.5% required immunomodulatory medications at 14 weeks compared to universal requirement in the formula-only group. At 24 weeks, 25% of patients remained in remission without immunomodulators, and among those who avoided immunomodulatory therapy throughout, 90% maintained remission [87]. These observations support this dietary strategy as a viable long-term management for appropriately selected mild-to-moderate disease patients when specialized IBD dietitian support is available.

More recently, an Italian open-label randomized trial by Pasta et al. compared CDED + PEN (tailored to 10–20% of energy needs) with a Mediterranean diet over 24 weeks in 45 adults with mild-to-moderate CD on stable medical therapy [85]. Remission rates were significantly higher in the CDED group at both week 12 (70.8% vs. 38.1%, *p* = 0.027) and week 24 (79.2% vs. 42.9%, *p* < 0.0001), with significantly lower HBI scores and higher remission rates than controls. Despite these clinical benefits, no significant reductions were observed in CRP or calprotectin, although the erythrocyte sedimentation rate improved only in the CDED group [85]. Notably, bioelectrical impedance analysis revealed that the CDED was associated with a significant decrease in BMI (25.8 to 24.5 kg/m^2^, *p* = 0.047), driven by a reduction in fat mass (18.2% to 15.5%, *p* < 0.0001) while fat-free mass and body cellular mass significantly increased at 12 weeks (*p* = 0.001 and *p* = 0.042, respectively) and remained stable at 24 weeks [85]. These body composition findings are particularly relevant, as they suggest that CDED may improve nutritional quality beyond simple weight change, a consideration of clinical importance given the high prevalence of sarcopenia in IBD [88].

Beyond its established role as induction therapy, CDED + PEN has shown promise as an adjunctive strategy in patients experiencing secondary loss of response to biologic therapies. Sigall Boneh et al. reported 60% clinical remission at 6 weeks in 21 patients (11 adults and 10 children) failing biologic therapy [89]. In a larger real-world pediatric cohort, Scarallo et al. treated 26 children with CD experiencing disease relapse despite maintenance biologic therapy with CDED + PEN for 8 weeks; 73% achieved clinical remission, 62% normalized CRP, and 31% normalized calprotectin, with remission rates similar regardless of whether the diet was initiated at diagnosis or after loss of response [90]. A small open-label randomized trial by Arcucci et al. in 21 children in clinical remission on biologics but with elevated calprotectin (>250 µg/g) found that calprotectin decreased by more than 50% in 82% of the CDED + PEN group versus 20% in the control group (*p* = 0.005), and none in the CDED group required biologic intensification compared with eight in the control group (*p* = 0.005) [91]. These findings support the concept of combining dietary and pharmacological approaches and highlight the potential of CDED + PEN as an add-on therapy to biologics, an area that warrants further investigation [88].

#### 4.2.2. Mechanism of Action and Microbiome Effects

Microbial analysis revealed distinctive patterns with the combined dietary approach. Levine observed reductions in *Haemophilus*, *Veillonella*, *Anaerostipes*, and *Prevotella* alongside increases in *Oscillibacter* and *Roseburia* [17]. Notably, these microbial shifts persisted throughout the twelve-week observation period in diet-following patients, whereas formula-only patients reverted to their baseline microbial profiles upon solid food reintroduction, suggesting that specific whole foods may sustain favorable microbial changes more durably than formula alone. This difference is clinically significant: while EEN cannot be maintained long-term due to palatability and practical constraints, CDED can be continued as maintenance therapy, allowing for more prolonged and sustained microbiome modifications [92]. At broader taxonomic levels, increases in *Firmicutes*, *Clostridiales*, *Clostridia*, and *Ruminococcus* occurred alongside decreases in Gammaproteobacteria, though SCFAs and bile acids showed no changes. Complementary metabolomic analysis of tryptophan pathways in the same cohort demonstrated that a reduction in kynurenine pathway metabolites (kynurenine and quinolinic acid) was strongly associated with induced remission with both CDED + PEN and EEN, while increases in serotonin pathway metabolites (melatonin, N-acetylserotonin, and 5-OH-tryptophan) were selectively associated with sustained remission at week 12 [76]. Importantly, patients failing to sustain remission showed no significant changes in these metabolites compared to baseline, and the ratios of kynurenine-to-melatonin and quinolinic acid-to-melatonin performed remarkably well as biomarker signatures for sustained remission with both dietary interventions [76].

Comprehensive metagenomic sequencing of 54 pediatric patients achieving remission demonstrated compositional shifts toward healthy control patterns, with decreased *Proteobacteria* (especially *Escherichia*, *Burkholderiales*, *Klebsiella*) and increased *Firmicutes* (*Clostridiales* including *Roseburia*, *Oscillibacter*, *Ruminococcus*), although E. coli remained elevated at twelve weeks [78]. Patients following the combined dietary approach maintained these corrections, while those on formula alone experienced dysbiosis relapse after dietary liberalization. Unsupervised Bayesian clustering identified two metabotypes: M1 (high *Bacteroidetes*/*Firmicutes*, low *Proteobacteria*, elevated SCFAs synthesis pathways) present in all healthy controls, and M2 characterizing disease states. The M1 metabotype contribution increased from 48% at baseline to 74% at week twelve during successful therapy [78]. Importantly, although the shift toward M1 indicates increased genomic capacity for SCFA synthesis, measured fecal concentrations did not increase correspondingly. This dissociation between genetic potential and metabolic output suggests that the clinical benefits observed during this timeframe were driven primarily by pathobiont reduction (particularly Proteobacteria) rather than by enhanced SCFA production. Whether longer intervention periods might eventually translate this increased genomic capacity into measurable metabolite changes remains to be determined. Primary bile acids decreased only with formula therapy, while secondary bile acids were unaffected by either intervention.

### 4.3. Tasty and Healthy

An innovative whole-food dietary approach known as Tasty and Healthy has been developed as an alternative therapeutic option for CD management [93]. This nutritional strategy eliminates processed items, gluten-containing products, red meat, and most dairy products while permitting plain yogurt, notably without requiring the formula-based supplementation that characterizes traditional exclusive enteral nutrition protocols.

#### 4.3.1. Clinical Effectiveness

The physician-blinded randomized controlled TASTI-MM study examined this dietary intervention against conventional exclusive enteral nutrition across an eight-week treatment period in a pediatric and young adult population (83 participants with mean age 14.5 years) experiencing mild-to-moderate disease activity. Clinical efficacy assessment through weighted Pediatric CD Activity Index and CD Activity Index measurements revealed comparable therapeutic effectiveness between the approaches, with symptomatic remission achieved in 56% versus 38% on intention-to-treat analysis and 67% versus 76% in per-protocol analysis [94]. Biochemical parameters, including C-reactive protein, erythrocyte sedimentation rate, and FC, demonstrated similar improvements across both interventions, with calprotectin values below 250 μg/g attained in 34% of whole-food diet patients compared to 33% receiving formula nutrition, while MINI scores under 8 occurred in 44% versus 31%, respectively. The most striking distinction emerged in treatment tolerance and adherence, where the whole-food approach achieved 88% compliance versus 52% for formula-based therapy (adjusted odds ratio 7.7, *p* < 0.001) [94].

#### 4.3.2. Mechanism of Action and Microbiome Effects

Sophisticated shotgun metagenomic analysis generating average sequencing depths of 96.8 million reads per sample at baseline and follow-up timepoints uncovered substantial microbiological differences despite equivalent clinical outcomes [94]. The whole-food dietary pattern promoted significantly enhanced alpha diversity as quantified by the Shannon and Chao1 index, with critically important progressive increases from baseline measurements, contrasting with formula nutrition, which failed to demonstrate such diversity improvements [94]. Microbiome compositional stability between sequential sampling periods proved superior with the whole-food intervention [94]. Species-level taxonomic profiling revealed that formula-based nutrition was associated with significant expansion of the inflammation-associated organism *Ruminococcus gnavus* at both intermediate and final timepoints, whereas the whole-food diet enriched beneficial commensal species, including *Faecalibacterium prausnitzii* and *Bacteroides uniformis* [94]. These microbiological observations suggest that while both nutritional strategies achieve similar short-term clinical remission rates, the whole-food approach cultivates a more diverse, stable, and health-associated intestinal microbial ecosystem compared to the restricted taxonomic composition observed with formula-based therapy [94]. Further investigation into sustained clinical efficacy, mechanistic understanding of microbiome-mediated therapeutic effects, and potential utility for maintenance treatment remains necessary to establish this dietary strategy’s optimal position within CD therapeutic algorithms [93].

### 4.4. CD-TREAT

Svolos and colleagues introduced an innovative dietary approach called CD-TREAT, which employs ordinary foods to replicate the nutritional profile and therapeutic mechanisms of EEN while offering improved palatability for patients with CD [95]. This carefully constructed diet matches enteral formulas in macronutrient distribution (protein, carbohydrate, and fat proportions) and micronutrient content, while also aligning with formulas in terms of fatty acid composition and fiber levels. The regimen eliminates gluten, lactose, and alcohol, and maintains restricted quantities of starches and fiber to more closely mirror commercial enteral products.

#### 4.4.1. Clinical Effectiveness

The culminating pilot clinical trial enrolled five pediatric patients with active CD [95], of whom four completed the eight-week intervention protocol. Results proved encouraging, with all four completers achieving clinical remission and demonstrating statistically significant improvements in disease activity indices. Objective markers of inflammation also improved substantially, with FC levels decreasing by an average of 55%. Participants consistently reported that CD-TREAT was more palatable and manageable than traditional EEN, potentially addressing a major barrier to dietary therapy adherence.

#### 4.4.2. Mechanism of Action and Microbiome Effects

The research team conducted a comprehensive three-phase investigation to evaluate CD-TREAT’s effectiveness. Initial testing in 25 healthy adults demonstrated that this food-based intervention produced comparable alterations in fecal microbiome composition and metabolite profiles to those observed with EEN, including similar modifications in bacterial diversity, SCFAs, and pH levels [95]. These preliminary observations showed moderate-to-strong correlations for changes in operational taxonomic units and genera between the two dietary approaches. Subsequent validation in HLA-B27 transgenic rats experiencing intestinal inflammation revealed that both interventions similarly diminished ileitis severity while inducing parallel shifts in gut microbial communities.

Despite these promising findings, important limitations merit acknowledgment. First, the specific food composition of CD-TREAT has not been fully published, limiting the ability of other centers to replicate the intervention. Second, microbiome analysis data in the IBD population who underwent this intervention are lacking.

## 5. Diets for Induction and Maintenance of Remission in Ulcerative Colitis

### 5.1. Curcumin and QingDai

Curcumin, the active compound of turmeric (Curcuma longa), and QingDai (also known as indigo naturalis) are plant-based compounds that have gained attention as potential therapeutic agents in UC. The combination of these two herbal extracts (CurQD) has been studied for its synergistic anti-inflammatory effects. The CurQD formulation used in clinical trials employs enteric-coated capsules designed to reduce systemic absorption and increase local mucosal exposure in the colon.

#### 5.1.1. Clinical Effectiveness

Curcumin as an adjunctive therapy to mesalamine has demonstrated efficacy in inducing remission in mild-to-moderate UC across multiple RCTs. Lang et al. randomized 50 mesalamine-treated patients with active UC who had not responded to maximum-dose mesalamine therapy to receive curcumin 3 g/day or placebo for one month. Clinical remission (Simple Clinical Colitis Activity Index (SCCAI), ≤2) was achieved in 53.8% of curcumin-treated patients compared with 0% in the placebo group (*p* = 0.01; OR 42), with clinical response in 65.3% versus 12.5% (*p* < 0.001) and endoscopic remission in 38% versus 0% (*p* = 0.043) [96]. The combination of curcumin with QingDai (CurQD) has shown superior efficacy in moderate-to-severe UC. In a randomized, double-blind, placebo-controlled trial, Ben-Horin et al. enrolled 42 patients with active UC (SCCAI ≥ 5, Mayo endoscopic subscore ≥ 2) and randomized them 2:1 to enteric-coated CurQD 3 g/day or placebo for eight weeks [97]. The co-primary outcome (clinical response plus objective response) was achieved in 43% of CurQD patients versus 8% in the placebo group (*p* = 0.033). Clinical response was observed in 85.7% versus 30.7% (*p* < 0.001), clinical remission in 50% versus 8% (*p* = 0.01), and endoscopic improvement in 75% versus 20% (*p* = 0.036). Fecal calprotectin reduction of at least 50% occurred in 46.4% versus 15.4% (*p* = 0.08). Responding patients who continued curcumin alone as maintenance therapy showed sustained clinical response in 93%, clinical remission in 80%, and clinic-biomarker response in 40% at week 16. Real-world data from a multicenter retrospective cohort of 88 patients with active UC (50% biologic/small molecule experienced) confirmed these findings [98]. Clinical remission was achieved in 46.5% and clinical response in 60.2%, with median SCCAI decreasing from seven to two (*p* < 0.0001). Median fecal calprotectin decreased from 1000 μg/g to 75 μg/g (*p* < 0.0001). Among biologic-experienced patients, clinical remission and response rates were 39.5% and 58.1%, respectively, suggesting efficacy independent of prior biologic failure. A pediatric multicenter retrospective study similarly demonstrated the effectiveness of CurQD in children with active UC [99]. Evidence supports a potential role for curcumin in UC maintenance. Hanai et al. found that curcumin 2 g/day combined with mesalamine was more effective than placebo at maintaining remission at six months (relapse rate 4% versus 18%), though this effect was not sustained at twelve months (22% versus 32%) [100]. Evidence for curcumin in CD is limited and less favorable. A RCT evaluating curcumin 3 g/day as an adjunct to azathioprine for preventing postoperative recurrence in CD found no benefit compared with placebo (recurrence 67.7% versus 58.1%, *p* = 0.60) [101,102]. Concerningly, higher rates of severe disease recurrence occurred in the curcumin group (54.8% versus 25.8%, *p* = 0.034), leading the ECCO 2025 Consensus to recommend against curcumin for CD maintenance [40]. However, emerging real-world data suggest the potential benefit of CurQD in active CD. A multicenter retrospective study of 30 patients with active CD (43% biologic-experienced) reported clinical remission in 48% and clinical response in 76%, with median Patient-Reported Outcome 2 (PRO2) score decreasing from 15 to 4 (*p* < 0.001) [103]. Biomarker remission and response were achieved in 55% and 75%, respectively, with fecal calprotectin decreasing from 639 to 138 μg/g (*p* = 0.001). A signal for better efficacy was observed in patients with colonic disease involvement (L2/L3) compared with isolated small bowel disease (L1), with biomarker remission in 73% versus 33% (*p* = 0.08). Drug retention at eight months median follow-up was 84% among responders. Adverse events associated with curcumin and QingDai are generally mild but warrant monitoring. Hepatotoxicity has been reported with curcumin, particularly with turmeric extracts, and modest liver transaminase elevations (up to three times the upper limit of normal) have occurred in 4–5% of patients, typically resolving with dose reduction or continuation. Headaches occur in approximately 2–3% of patients and are usually self-limiting. QingDai has been associated with rare cases of reversible pulmonary arterial hypertension when used at high doses for prolonged periods, predominantly reported in Japanese populations. Other rare adverse events include intussusception and ischemic colitis. Curcumin may interact with medications including anticoagulants and diabetes drugs and may modulate estrogen receptors. Medical supervision with monitoring of liver biochemistry is recommended, particularly for long-term and high-dose use.

#### 5.1.2. Mechanism of Action and Microbiome Effects

Curcumin exerts anti-inflammatory effects through multiple mechanisms, including inhibition of nuclear factor-kappa B (NF-κB) signaling, reduction in pro-inflammatory cytokines (TNF-α, IL-1β, IL-6), and antioxidant activity. QingDai contains indigo and indirubin as active compounds, which function as potent AhR agonists [104,105]. The combination may provide synergistic effects through complementary pathways.

The therapeutic mechanism of CurQD appears to involve the activation of the aryl hydrocarbon receptor (AhR) pathway, a ligand-activated transcription factor involved in mucosal epithelial reconstitution and immunoregulation. In the RCT by Ben-Horin et al., CurQD treatment uniquely resulted in up-regulated expression of cytochrome P450 1A1 (CYP1A1) in rectal mucosa, a marker of AhR pathway activation [97]. This upregulation was not observed in patients receiving placebo, mesalamine, or biologic drugs, suggesting a distinct mechanism of action. AhR signaling has been implicated in promoting intestinal barrier integrity, regulating immune responses, and suppressing inflammation through effects on innate lymphoid cells and other immune cell populations [106].

The observation that CurQD may be more effective in colonic than small bowel CD prompted investigation into differential AhR expression along the gut axis. Analysis of the Genotype-Tissue Expression (GTEx) database revealed comparable mRNA expression of AhR in the human colon and small bowel mucosa, both significantly elevated compared with whole blood. This suggests that the signal for better efficacy in colonic disease likely reflects the generally more treatment-resistant nature of small bowel CD rather than differential receptor expression.

In IBD specifically, curcumin’s effects on the microbiome have been studied in the context of UC. Curcumin reduces disease activity and endoscopic scores while increasing beneficial bacteria (*Lactobacillus*, *Bifidobacterium*) and decreasing pro-inflammatory bacterial species. In preclinical models, curcumin has shown efficacy in suppressing mucosal expression of inflammatory mediators, inhibiting the proliferation of colon cancer cells, and inducing apoptosis [107]. The promotion of SCFA-producing bacteria contributes to intestinal mucosal protection and mitigation of the inflammation associated with intestinal diseases. The bidirectional curcumin–microbiota interaction also influences curcumin’s bioavailability, as gut bacteria generate colonic metabolites with potent pharmacological activities. A meta-analysis of 15 animal studies demonstrated that indigo naturalis significantly reduced pro-inflammatory cytokines while increasing IL-10, partly through microbiota–butyrate axis modulation and increased abundance of *Ruminococcus* and *Butyricicoccus* [108].

### 5.2. Mediterranean Diet

The MD is characterized by an abundance of fruits, vegetables, legumes, whole grains, olive oil, and nuts, with moderate intake of fish and wine and minimal consumption of red meat and processed foods [109]. This dietary pattern has been shown to promote a diverse and beneficial gut microbiota composition [110] and represents one of the most comprehensively studied patterns with potential benefits extending beyond IBD to include cardiovascular health and metabolic disorders [111,112].

#### 5.2.1. Clinical Effectiveness

In IBD populations, observational studies have linked adherence to the MD with lower inflammatory markers and better disease-related quality of life. Chicco et al. found that adherence to the MD correlated with lower C-reactive protein levels and higher IBD questionnaire scores in a prospective study of 142 patients (both active and quiescent) [113]. After six months, both patients with UC and patients with CD adhering to the MD demonstrated lower rates of active disease, inflammatory biomarker elevation, and improved quality of life. In patients with active IBD, the MD has been shown to reduce disease activity and markers of inflammation, including fecal calprotectin (FC) and C-reactive protein [114,115].

In Canadian Patients with UC in remission, the MD decreased FC and increased SCFA production compared to a standard Canadian diet [116]. A sub-analysis of participants with UC showed that those who adhered to the MD were less likely to experience flares, had lower FC, and had reduced need for corticosteroids, with a median time to relapse of fourteen months compared to 5.5 months in non-adherent patients. According to the ECCO 2025 Consensus, the MD can be considered for maintenance of remission in UC [40].

#### 5.2.2. Mechanism of Action and Microbiome Effects

In patients with IBD, adherence to the MD appears to modulate the microbiota favorably through several mechanisms. The diet increases anti-inflammatory bacteria such as *Faecalibacterium prausnitzii*, *Roseburia*, and *Bifidobacterium* [110], all recognized as butyrate producers. Concurrently, it reduces pro-inflammatory taxa associated with dysbiosis, including *Proteobacteria* [110]. The high levels of dietary fibers and polyphenols provided by the MD nourish beneficial bacteria and strengthen the intestinal barrier [117,118,119]. Additionally, this dietary pattern modulates tryptophan metabolism, promoting microbial production of the aryl hydrocarbon receptor ligands involved in Glucagon-like peptide-1 (GLP-1) secretion and intestinal integrity [120,121,122]. Through these mechanisms, the MD represents a dietary model capable of promoting and maintaining an eubiotic microbiota, increasing anti-inflammatory SCFA production, and reducing IBD-associated dysbiosis.

The MD exerts favorable effects on gut microbiota composition and metabolic activity. Studies have demonstrated higher levels of SCFAs with greater adherence to the MD, with positive correlations between plant-based food intake and abundance of *Roseburia*, *Lachnospira*, and *Prevotella* [118,123,124]. In patients with CD, adherence to the MD for six weeks resulted in a shift toward more normalized gut microbe composition, characterized by increases in *Bacteroidetes* and *Clostridium* clusters along with decreases in *Proteobacteria* and *Bacillaceae* [124]. Furthermore, increases in *Faecalibacterium prausnitzii*, *Roseburia*, *Lachnospiraceae*, and *Clostridiales* taxa were observed, alongside decreases in *Ruminococcus torques* and *Ruminococcus gnavus*. One study found a decrease in total bile acids with adherence to the MD, and this decrease was associated with reduced *Bilophila wadsworthia abundance* [124]. Haskey found that species primarily belonging to the phylum *Firmicutes*, including *Ruminococcus*, *Flavonifractor*, *Clostridium*, *Lactococcus*, and *Blautia* species, were most positively associated with MD [116]. Strauss and colleagues used weighted gene co-expression network analysis to identify gut microbiome features mediating the relationship between MD adherence and inflammation in patients with UC [125]. Three bacterial taxa (*Faecalibacterium prausnitzii*, *Dorea longicatena*, and *Roseburia inulinivorans*), along with a metabolite cluster (benzyl alcohol, 3-hydroxyphenylacetate, 3-4-hydroxyphenylacetate, and phenylacetate), demonstrated strong mediating effects between diet and FC levels. The most comprehensive mechanistic investigation followed 271 patients with newly diagnosed CD prospectively for 24 months, demonstrating that MD adherence was associated with noncomplicated disease course and inversely correlated with disease activity index, inflammatory markers, and microbial dysbiosis index (all *p* < 0.05) [126]. Mechanistically, MD correlated positively with beneficial butyrate-producing taxa, including *Faecalibacterium* and plant-derived anti-inflammatory metabolites, while inversely correlating with pathobionts (*Escherichia coli*, *Ruminococcus gnavus*), pro-inflammatory tryptophan metabolites, ceramides, and primary bile acids. Increasing adherence over time correlated with reduced inflammatory markers, demonstrating the feasibility and potential of dietary modification as a disease-modifying strategy in newly diagnosed CD.

The MD shows increased SCFAs with decreased branched-chain fatty acids, decreased bile acids, and decreased tryptophan with increased indole-3-propionic acid, indole-3-lactic acid, and indole-3-acetic acid [69].

### 5.3. Reduction in Red and Processed Meat

Plant-based dietary patterns, ranging from vegetarian to vegan approaches, have garnered attention for their potential anti-inflammatory effects and positive impact on gut microbiota composition [127,128]. These diets emphasize whole plant foods rich in fiber, polyphenols, and antioxidants while limiting or excluding animal products and processed foods.

#### 5.3.1. Clinical Effectiveness

The ECCO 2025 Consensus states there is insufficient evidence to recommend plant-based diets for the induction or maintenance of remission in CD or UC. However, plant-based dietary patterns align with general healthy eating recommendations [40]. Nevertheless, a systematic evaluation examining twenty-three investigations with 2304 total participants indicated that most research documented both the safety and effectiveness of plant-based approaches in attenuating disease activity and preserving remission states, albeit with recognized methodological constraints [129]. Particularly compelling evidence emerged from prospective semi-vegetarian diet trials in CD management, where 94% of adherent patients maintained remission versus merely 33% consuming omnivorous diets [130]. This semi-vegetarian protocol emphasized daily consumption of legumes, vegetables, fruits, potatoes, and yogurt while excluding sweets and confectionery products, delivering 30 to 35 g of predominantly plant-derived fiber per 2000 kilocalories. Parallel favorable outcomes were documented in UC populations following comparable dietary frameworks [131].

#### 5.3.2. Mechanism of Action and Microbiome Effects

Mechanistically, these dietary interventions appear to operate through microbiome modulation. Plant-predominant eating patterns have been associated with the expansion of numerous commensal taxa, including *Roseburia*, *Ruminococcus*, *Streptococcus thermophilus*, *Bacteroides thetaiotaomicron*, *Clostridium clostridioforme*, *Faecalibacterium prausnitzii*, *Prevotella*, and *Lachnospira*, while simultaneously reducing *Clostridium* cluster XIVa, *Alistipes*, and *Proteus mirabilis* populations. A three-month ovo-lacto-vegetarian intervention documented increases in *Roseburia*, *Ruminococcus*, and *Streptococcus thermophilus* alongside decreases in *Alistipes* and *Proteus mirabilis* [132]. At higher phylogenetic classifications, both *Bacteroides* and *Prevotella* within *Clostridium* cluster XIV demonstrated expansion. Additional beneficial taxa enrichment has been reported, encompassing Bacteroidetes phylum members, *Eubacterium rectale*, *Bifidobacterium*, and *Lactobacillus*, which collectively contribute anti-pathogenic, anti-inflammatory, and cardiovascular protective properties [133].

Functionally, vegetarian dietary patterns correlate with elevated SCFAs, including butyrate, propionate, and acetate, relative to omnivorous consumption, with these metabolic shifts positively associated with *Prevotella* and Firmicutes members such as *Roseburia* and *Lachnospira* [134]. Concurrently, plant-exclusive diets demonstrate reduced abundances of potentially detrimental organisms, including *Clostridium*, *Enterococcus*, and bile-resistant species like *Bacteroides* and *Bilophila,* typically associated with elevated meat and fat consumption [135,136]. Vegans show diminished fecal bile acid concentrations compared to omnivores, while vegetarians exhibit reduced serum kynurenine metabolites alongside elevated tryptophan levels. Generally, vegan and vegetarian populations display enhanced microbial diversity and temporal stability (α-diversity), both considered markers of intestinal wellness [137].

However, interpretive caution remains warranted. Cross-sectional analyses and systematic reviews have failed to identify consistent microbial compositional signatures distinguishing vegetarian or vegan diets from omnivorous patterns [138], revealing substantial inter-individual variation. Researchers emphasize that diet-induced microbial alterations following brief interventions tend to be modest and reversible, whereas sustained multi-year dietary adherence produces more durable ecosystem modifications [139]. The therapeutic mechanisms likely involve postbiotic metabolites, including SCFAs, phytochemical compounds, and vitamins, which collectively exert anti-inflammatory, antioxidant, and cardioprotective actions. This mechanistic complexity, combined with marked individual variability in response patterns, explains the heterogeneous outcomes observed across different study populations [127,128].

## 6. Diets for IBS-like Symptoms in IBD

### 6.1. Low-FODMAP Diet

The Low-FODMAP (LF) diet substantially influences gut microbiota composition through its restriction of fermentable carbohydrates, which are potent modulators of microbial ecology [140]. The LF diet restricts short-chain carbohydrates that are poorly absorbed in the small intestine and rapidly fermented by gut bacteria, thereby increasing intestinal water content and gas production. High-FODMAP foods commonly eliminated include onions, garlic, wheat, legumes, certain fruits such as apples and pears, and lactose-containing dairy products. These foods are typically omitted for up to eight weeks, followed by systematic reintroduction to assess individual tolerance [109].

#### 6.1.1. Clinical Effectiveness

A substantial proportion of IBD patients in remission experience persistent irritable bowel syndrome-like symptoms, with prevalence estimates ranging from 35% to 46% across various studies [141,142]. A recent comprehensive review by Damianos et al. (2026) highlighted that small intestinal microbial dysbiosis (SIMD, an updated term encompassing bacterial, archaeal, and fungal overgrowth) is prevalent in IBD even during remission, with positive breath tests reported in up to 46.6% of patients with CD in remission, and that the presence of SIMD was associated with a significantly higher rate of subsequent clinical relapse [143]. In IBD populations, the primary relevance of the LF diet lies in managing overlapping functional symptoms rather than addressing intestinal inflammation directly [144]. For these individuals, FODMAP restriction can provide meaningful symptom relief without affecting underlying disease activity [145]. Prince et al. demonstrated that fermentable carbohydrate restriction improved functional gastrointestinal symptoms in clinical practice, with particular benefits observed for abdominal pain, bloating, and altered bowel habits [146]. An RCT involving eighty-nine adult IBD patients in remission or with mild-to-moderate disease showed that six weeks of LF diet resulted in significant improvements in quality of life and reduction in concomitant irritable bowel syndrome symptoms compared to a standard diet [146]. Similarly, a prospective study with fifty-five IBD subjects demonstrated that the LF diet reduced clinical disease activity in patients with mild disease or in remission after six weeks compared with standard dietary management [147]. Notably, in a small study of nine patients with CD in remission, the LF diet affected gastrointestinal symptoms while increasing the relative abundance of butyrate-producing bacteria from *Clostridium* cluster XIVa and the mucus-associated species *Akkermansia muciniphila* [148].

#### 6.1.2. Mechanism of Action and Microbiome Effects

The microbiological consequences of the LF diet include significant alterations in fecal microbial composition. Cox’s analysis of stool samples following LF diet intervention revealed a significant decrease in *Faecalibacterium prausnitzii* and changes in three *Bifidobacterium* species: decreased *Bifidobacterium adolescentis* and *Bifidobacterium dentium* with increased *Bifidobacterium longum* [149]. In an Australian study, Halmos demonstrated a decreased abundance of *Clostridium* cluster XIVa and *Akkermansia muciniphila* alongside increased *Ruminococcus torques* in the LF diet compared to a standard diet [69]. The LF diet also showed lower bile acid levels and higher indole-3-propionic acid than a placebo diet [69]. These findings underscore that prolonged FODMAP restriction may negatively impact gut microbiota diversity by reducing the intake of fermentable fibers that serve as prebiotics, necessitating a phased approach with systematic reintroduction of tolerated foods following initial restriction [150]. Furthermore, the restriction of foods containing beneficial compounds such as fructans, which have been suggested to exert therapeutic effects on CD by modifying dendritic cells to release interleukin-10 and increasing *Bifidobacteria* populations, represents a potential limitation of this dietary approach [151,152].

To address these limitations, a Mediterranean LF diet has recently been proposed as a sequential and personalized strategy [153]. This approach integrates the elimination and reintroduction phases of the traditional LF diet with a long-term maintenance phase incorporating MD principles, including non-fermentable fibers, tolerated legumes, olive oil, fish, and nuts [154]. Moreover, Zhang and colleagues investigated the relationship between dietary patterns and gut microbiota in CD patients in remission, finding that patients consuming a non-diversified diet had lower *Faecalibacterium* and higher *Escherichia*/*Shigella* relative abundances compared to those on a diversified diet [155]. Following a 12-week diversified dietary intervention, the non-diversified diet group demonstrated increased *Faecalibacterium* abundance and microbial composition that resembled the diversified diet group. These results demonstrate that dietary patterns shape the microbial dysbiosis signature in CD towards a more balanced microbial community.

## 7. Diets with Insufficient Evidence or Not Recommended

### 7.1. Carbohydrates Modified Diets

The Specific Carbohydrate Diet operates on the theoretical premise that complex carbohydrates, including disaccharides and polysaccharides, undergo incomplete absorption within inflamed intestinal tissue and subsequently provide nutritional substrates for pathogenic microorganisms, potentially amplifying inflammatory processes [156]. The regimen mandates the elimination of all cereal grains and derived products, refined sweeteners and confections containing high-fructose corn syrup, high-lactose dairy products, starchy vegetables, and all sugars with the singular exception of honey.

#### 7.1.1. Clinical Effectiveness

Within pediatric cohorts, retrospective evidence indicates that roughly 36% of patients attained clinical remission between one and three months following dietary implementation [157]. A prospective investigation involving ten children with active CD utilized capsule endoscopy to assess mucosal healing, revealing that 40% achieved this endpoint by twelve weeks as defined by a Lewis Score under 135, while 80% demonstrated substantial mucosal improvement relative to baseline measurements [158]. In adult populations, an RCT directly comparing this dietary approach with an MD in patients with CD found equivalent effectiveness [159]. Additional observational evidence includes a case series documenting clinical improvement in 33 IBD patients, with 42% reaching complete remission [160]. Lorenz-Meyer and colleagues conducted an RCT comparing omega-3 fatty acids and a carbohydrate-reduced regimen (84 g daily) against placebo for maintaining remission in 204 CD patients [92]. While omega-3 supplementation provided no benefit over placebo (both 30% maintaining remission at one year), adherent patients following the low-carbohydrate protocol demonstrated a significant advantage (53% maintaining remission, *p* = 0.023), though intention-to-treat analysis diminished this benefit (40% versus 30% for placebo), underscoring adherence’s critical importance. Ritchie and colleagues in 1987 published a controlled multicenter trial comparing unrestricted sugar with low-fiber intake against low-sugar with high-unrefined carbohydrate consumption in 352 patients with inactive or mildly active CD, finding no discernible differences in clinical outcomes after two years [161]. An RCT demonstrated that the MD was as effective as the Specific Carbohydrate Diet in adults with CD, with both approaches showing similar rates of symptomatic remission and reductions in inflammatory biomarkers [159].

#### 7.1.2. Mechanism of Action and Microbiome Effects

Microbiological investigations have documented alterations in fecal microbial composition following dietary implementation, though specific changes demonstrate considerable inter-individual variability. Microbiome analysis revealed increased abundance of *Roseburia*, *Lachnospiraceae*, and *Blautia* species, along with *Faecalibacterium prausnitzii*, *Anaerobutyricum*, and *Eubacterium eligens*, though these alterations varied substantially between individuals without clear associations with remission status [162]. Comparative research contrasting a low-residue diet with this carbohydrate-restricted approach found enhanced microbial diversity following the latter versus decreased diversity with the former, accompanied by increased *Faecalibacterium prausnitzii* and *Clostridium* species in those following the carbohydrate restriction [163]. In pediatric patients assessed over two weeks, most demonstrated decreased *Proteobacteria*, with *Bacteroides* and *Parabacteroides* showing the greatest reductions, while *Eubacterium*, *Ruminococcus*, and *Subdoligranulum* exhibited the largest increases [164]. Notably, Lewis observed that this dietary intervention resulted in diminished *Faecalibacterium prausnitzii* abundance compared with MD, alongside elevated *Enterobacteriaceae* family members and reduced *Eubacterium eligens* and *E. rectale* [46], representing a potentially concerning reduction in beneficial butyrate-producing bacteria

Despite considerable patient popularity, existing evidence does not support this regimen as first-line dietary therapy for IBD management. Current ECCO guidelines state that insufficient evidence exists to recommend this dietary approach for inducing or maintaining remission in either CD or UC [163].

### 7.2. Fiber Diets

The optimal approach to dietary fiber in IBD has undergone substantial reconsideration in recent years [61]. Historically, clinicians broadly recommended low-fiber or low-residue diets to patients with IBD, based on the premise that fiber restriction might reduce stool output and alleviate gastrointestinal symptoms during active disease, particularly diarrhea and abdominal pain. This conventional approach distinguished between insoluble fibers such as cellulose, hemicellulose, and lignin, and soluble fibers including pectins, gums, and mucilages that form viscous gels when combined with water. However, emerging evidence has highlighted the beneficial effects of dietary fiber fermentation by gut bacteria, which generates SCFAs such as butyrate that fuel colonocytes, strengthen intestinal barrier integrity, and mediate anti-inflammatory pathways.

#### 7.2.1. Clinical Effectiveness

The therapeutic potential of prebiotic interventions in IBD has been examined through a comprehensive systematic review encompassing 17 studies, with 13 contributing to quantitative analysis [165]. For inducing remission in UC, the fructooligosaccharide kestose demonstrated efficacy with a relative risk of 2.75 (95% CI 1.05–7.20, n = 40), whereas both oligofructose-enriched inulin and lactulose failed to show benefit. Germinated barley foodstuff showed a trend toward preventing relapse during UC maintenance (RR 0.40, 95% CI 0.15–1.03, n = 59). No prebiotics proved beneficial for either inducing or maintaining remission in CD, and oligofructose-enriched inulin actually increased adverse events (RR 3.10, 95% CI 1.54–6.21), predominantly flatulence and bloating. Hallert’s investigation of Patients with UC in remission consuming twenty grams of dietary fiber daily for twelve weeks revealed a 36% elevation in fecal butyrate concentration at four weeks, accompanied by marked improvements in gastrointestinal symptomatology, particularly abdominal pain, by study completion [166]. Notably, no colitis flares occurred throughout the intervention.

The traditional low-residue approach to IBD management contrasts sharply with emerging high-fiber strategies, though careful patient selection remains essential.

A preliminary investigation enrolled 25 patients with UC randomized to receive either 7.5 g/d (n = 12) or 15 g/d (n = 13) of oligofructose-enriched inulin across nine weeks [167]. Clinical response rates reached 77% in the high-dose cohort versus 33% in the low-dose group, with Mayo score modifications exhibiting a strong correlation with FC levels [40].

A prospective evaluation of seventy patients with active CD comparing low-residue versus unrestricted diets found no meaningful differences in symptom management, complication rates, hospitalization frequency, surgical interventions, or nutritional parameters [168].

Current international guidelines from the European Society for Clinical Nutrition and Metabolism (ESPEN) and ECCO now encourage adequate fiber consumption during the remission phase, while recommending restriction of insoluble fiber and texture adaptation primarily for patients with symptomatic intestinal strictures or stenosis, though robust data supporting even this logical approach remain limited. The nuanced understanding that not all fibers exert equivalent effects and that individual tolerance varies substantially depending on disease activity, phenotype, and underlying microbiome composition has prompted a shift away from blanket fiber avoidance toward more personalized recommendations.

#### 7.2.2. Mechanism of Action and Microbiome Effects

Gut bacteria transform dietary fiber into SCFAs through fermentation, a process carried out by various microbial taxa, including *Bacteroides*, *Bifidobacterium*, *Blautia*, members of Lachnospiraceae, and several Clostridia genera, such as *Faecalibacterium*, *Eubacterium*, *Roseburia*, and *Ruminococcus*. SCFAs fuel colonocytes, strengthen intestinal barrier integrity, regulate immune function, and mediate anti-inflammatory pathways through diverse mechanisms [169].

**Figure 3:**
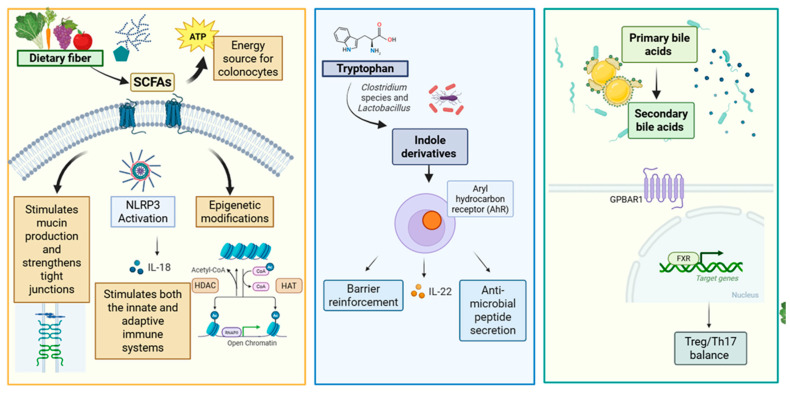
Molecular signaling pathways mediating the immunomodulatory effects of microbiota-derived metabolites in inflammatory bowel disease. Left panel: dietary fiber is fermented by gut bacteria into short-chain fatty acids (SCFAs), which activate NLRP3 inflammasome-mediated IL-18 release, modulate gene expression through histone deacetylase (HDAC) inhibition, stimulate mucin production and tight junction assembly, and serve as the primary energy source for colonocytes via β-oxidation and the tricarboxylic acid (TCA) cycle. Central panel: dietary tryptophan is metabolized by *Clostridium* species and *Lactobacillus* into indole derivatives that activate the aryl hydrocarbon receptor (AhR), promoting IL-22 secretion, barrier reinforcement, and antimicrobial peptide production. Right panel: primary bile acids are converted by gut bacteria into secondary bile acids that activate G protein-coupled bile acid receptor 1 (GPBAR1/TGR5) and farnesoid X receptor (FXR), modulating regulatory T cell *(Treg)/Th17* balance. Created in BioRender. Bertin, L. (2026) https://BioRender.com/ou2n3bt. Abbreviations: SCFAs, short-chain fatty acids; HDAC, histone deacetylase; HAT, histone acetyltransferase; NLRP3, NOD-, LRR- and pyrin domain-containing protein 3; AhR, aryl hydrocarbon receptor; FXR, farnesoid X receptor; GPBAR1, G protein-coupled bile acid receptor 1; TCA, tricarboxylic acid; MUC2, mucin 2; RNAPII, RNA polymerase II; MCT, monocarboxylate transporter; IκB, inhibitor of nuclear factor-kappa B.

At the molecular level, SCFAs exert their immunomodulatory effects through two principal signaling mechanisms (Figure 3). First, acetate, propionate, and butyrate serve as ligands for the G-protein coupled receptors GPR41 (also known as FFAR3) and GPR43 (FFAR2), which are expressed on intestinal epithelial cells, enteroendocrine cells, and various immune cells, including neutrophils, macrophages, and dendritic cells. Activation of GPR43 by acetate initiates potassium efflux-mediated hyperpolarization and subsequent calcium mobilization, which activates the NLRP3 inflammasome and downstream release of IL-18, thereby reinforcing epithelial barrier integrity [13]. Second, butyrate and propionate function as potent inhibitors of histone deacetylases (HDACs), an epigenetic regulatory mechanism that modulates gene expression in immune cells. HDAC inhibition by SCFAs suppresses NF-κB nuclear translocation and reduces expression of pro-inflammatory cytokines, including TNF-α, IL-1β, and IL-6, while simultaneously promoting the differentiation and expansion of regulatory T cells (Tregs) through enhanced Foxp3 expression. Butyrate further reinforces the mucosal barrier by stimulating mucus production from goblet cells and serving as the primary energy source for colonocytes, thereby supporting epithelial cell turnover and repair along the crypt–villus axis. Additionally, SCFAs and tryptophan metabolites produced by *Clostridium* species (clusters XIVa and IV) serve as dietary modulators of the aryl hydrocarbon receptor (AhR), a transcription factor that is characteristically under-expressed in colitis and that facilitates multiple anti-inflammatory actions, including the regulation of innate lymphoid cells and promotion of intestinal barrier integrity.

Molecular analysis via 16S rRNA pyrosequencing of matched fecal and mucosal specimens of patients undergoing a high fiber diet (15 g/d) revealed that the regimen increased fecal *Bifidobacteriaceae* and *Lachnospiraceae* while reducing mucosal *Bacteriaceae* [40]. However, these microbial compositional alterations showed no association with inflammatory markers. Mucosal *Faecalibacterium* and *Dialister* demonstrated negative correlations with inflammation, though these appeared to represent consequences rather than causative factors. Importantly, the higher dose enhanced total fecal SCFA production, with butyrate concentrations inversely correlating with Mayo scores.

### 7.3. Gluten-Free Diet

The gluten-free diet, which excludes all foods containing gluten, found in wheat, barley, rye, and triticale, represents the cornerstone of treatment for celiac disease and has garnered increasing interest as a potential therapeutic intervention for patients with IBS and IBD who experience non-celiac wheat sensitivity, a condition characterized by gastrointestinal symptoms such as bloating, abdominal pain, and altered bowel habits following gluten ingestion in the absence of celiac disease or wheat allergy [170,171,172,173,174]. Notably, the prevalence of celiac disease among IBD patients appears similar to that of the general population (approximately 0.5–1%), while the reported prevalence of non-celiac gluten sensitivity in IBD varies widely (4.9–27.6%) depending on study methodology and definitions employed [175]. A landmark prospective cohort study by Lopes and colleagues examined the relationship between long-term gluten intake and IBD risk in 208,280 US participants from the Nurses’ Health Study, NHSII, and Health Professionals Follow-up Study without celiac disease [169]. Over 5,115,265 person-years of follow-up, 337 CD cases and 447 UC cases were documented. Dietary gluten intake showed no association with risk of either CD (adjusted HR 1.16, 95% CI 0.82–1.64 for the highest vs. lowest quintile, P-trend = 0.41) or UC (adjusted HR 1.04, 95% CI 0.75–1.44, P-trend = 0.64). These findings remained consistent across various exposure time-windows and after adjustment for primary gluten sources, providing robust epidemiological evidence that gluten consumption does not influence IBD development in adults without celiac disease.

#### 7.3.1. Clinical Effectiveness

Despite lacking robust scientific support, gluten elimination has become increasingly common among individuals with IBD. Survey data from 1647 IBD patients revealed that approximately one-fifth had experimented with gluten restriction, with a majority subjectively reporting symptomatic relief and more than one-third noting what they perceived as decreased disease activity [176]. However, the first rigorously designed triple-blind RCT examining gluten restriction in twenty-six patients with mild-to-moderate UC over six weeks demonstrated no meaningful clinical benefits [177]. Neither erythrocyte sedimentation rate, C-reactive protein, IBD Questionnaire scores, nor Simple Clinical Colitis Activity Index values showed statistically significant improvements with dietary manipulation in either arm. FC levels increased non-significantly in both groups. The investigators concluded that gluten elimination showed no demonstrable effect on inflammatory biomarkers, quality of life metrics, or disease severity in this population, cautioning against premature adoption of gluten-free regimens for UC management [177].

The situation in CD presents a notable interpretive challenge. Several therapeutic diets demonstrating efficacy in CD—including EEN (inherently gluten-free), the CDED (which explicitly excludes gluten, dairy, processed foods, additives, and high-fat animal products), and the SCD (which restricts grains)—share gluten avoidance as a common feature. However, these interventions simultaneously eliminate multiple potentially inflammatory components, making it impossible to isolate gluten’s independent contribution to the observed clinical benefits. No RCT has specifically evaluated gluten restriction alone in CD, leaving the question of whether gluten per se contributes to disease activity—or whether its exclusion in successful dietary therapies is merely incidental—entirely unanswered. A substantial Swiss prospective cohort study tracking 1254 IBD patients found no meaningful distinctions between gluten-avoiding and gluten-consuming groups regarding disease activity, complications, hospitalization frequency, or surgical intervention rates [178]. Interestingly, patients following a gluten-free diet in this cohort reported worse overall psychological well-being compared to those not restricting gluten—a finding contrary to observations in non-IBD populations [175]. Furthermore, symptomatic improvements attributed to gluten elimination may actually stem from concurrent reduction in FODMAPs—wheat being a significant fructan source—that are frequently eliminated alongside gluten, complicating efforts to isolate gluten’s specific contribution [144,179]. A double-blind crossover trial in patients with non-celiac gluten sensitivity demonstrated significant symptom reduction with an LF diet, but was unable to reproduce gastrointestinal symptoms with dose-dependent gluten challenge, suggesting that FODMAPs rather than gluten itself may be the primary symptom trigger [175].

The ECCO 2025 Consensus explicitly states that current evidence is inadequate to support gluten avoidance as a therapeutic strategy for achieving or sustaining remission in IBD, recommending such dietary modifications exclusively for individuals with documented celiac disease or established non-celiac wheat sensitivity [40].

#### 7.3.2. Mechanism of Action and Microbiome Effects

Experimental animal investigations from Hamburg have suggested that gluten may exacerbate DSS-induced colitis in mice, while prolonged gluten-free feeding appears protective against colitis susceptibility. Notably, studies using germ-free animals indicate that these effects operate through microbiota-mediated mechanisms during extended gluten restriction, with 16S ribosomal RNA sequencing revealing altered bacterial population distributions in murine fecal samples [180]. Similarly, an exploratory investigation in PSC-IBD patients demonstrated that eight weeks of gluten elimination significantly reduced mucosal proinflammatory cytokine and chemokine expression while producing modest shifts in luminal microbial composition [6,181].

Despite these preliminary observations in animal models and select patient populations, comprehensive characterization of how gluten-free diets impact gut microbiota composition, diversity, and functional capacity in the broader IBD population remains lacking. Critically, disentangling the specific effects of gluten restriction from those of concurrent dietary modifications in multi-component exclusion diets represents a key research priority for understanding the mechanistic basis of any potential therapeutic effects.

### 7.4. Anti-Inflammatory Diets (IBD-AID, AIP)

Holistic nutritional strategies focusing on anti-inflammatory principles have emerged as potential therapeutic approaches in IBD management. These dietary frameworks prioritize minimally processed, whole food consumption while eliminating ingredients thought to exacerbate inflammatory responses [182]. One such intervention specifically designed for IBD patients incorporates substantial amounts of omega-3 polyunsaturated fatty acids, polyphenol-rich foods, and prebiotic compounds, while simultaneously limiting refined sugars, saturated lipids, and artificial additives [183].

#### 7.4.1. Clinical Effectiveness

Clinical investigations have yielded encouraging preliminary findings. Olendzki’s research documented that approximately 60% of participants maintaining adherence to anti-inflammatory dietary patterns experienced favorable or highly favorable clinical outcomes [182]. Subsequently, Keshteli’s work revealed that anti-inflammatory eating patterns prevented subclinical intestinal inflammation [184]. A small open-label investigation examining the Autoimmune Protocol diet, which extends anti-inflammatory dietary principles, enrolled nine patients with CD and six with UC. Results demonstrated improvements in quality of life measures, disease activity scores, and FC levels, with six of seven patients showing endoscopic improvement after eleven weeks [185].

Despite these theoretical benefits, current ECCO guidelines indicate that evidence remains insufficient to support recommending either this anti-inflammatory protocol or the Autoimmune Protocol diet for achieving or sustaining remission in either CD or UC [40].

#### 7.4.2. Mechanism of Action and Microbiome Effects

Microbiome analyses from IBD patients following this dietary intervention demonstrated enhanced representation of SCFA-synthesizing microorganisms within the *Clostridia* class, particularly *Roseburia hominis*, which predominated across all IBD participants and specifically within CD subjects. Patients with UC exhibited enrichment of *Faecalibacterium prausnitzii*, *Eubacterium eligens*, *Coprococcus catus*, and various *Bacteroides* species, whereas *Parabacteroides distasonis* showed consistent depletion throughout the IBD cohort during dietary intervention [186]. Functional capacity analysis revealed enhanced microbiome potential for synthesizing essential amino acids, degrading mannan polysaccharides, and oxidizing fatty acids. Participants completing the intervention demonstrated increased genetic capacity for butyrate synthesis, predominantly from *Clostridia* members, alongside enhanced acetate production specifically attributable to *Roseburia hominis* and *Eubacterium eligens*. Prebiotic, probiotic, and beneficial food consumption correlated with the expansion of *Clostridia* and *Bacteroides* populations, taxonomic groups frequently diminished in IBD populations [186].

Dietary component analysis revealed specific associations between food categories and microbial populations. Non-starchy vegetable consumption, along with nuts and seeds, positively correlated with proliferation of SCFAs-producing taxa, including *Roseburia hominis*, *Eubacterium eligens*, and *Faecalibacterium prausnitzii* [183]. Similarly, increased monounsaturated and omega-3 polyunsaturated fatty acid intake supported robust SCFAs-producing *Clostridia* and *Bacteroides* lineages [90]. Interestingly, elevated consumption of lean animal proteins, classified as beneficial within this dietary framework, demonstrated negative associations with *Roseburia hominis* abundance in UC but not CD patients [186].

### 7.5. Ketogenic Diet

Among dietary interventions gaining attention, the ketogenic approach warrants particular consideration given its metabolic distinctiveness. This nutritional strategy, initially conceived during the 1920s for managing refractory epilepsy [187], imposes severe carbohydrate restriction while substantially elevating fat consumption to 70–90% of calories and maintaining moderate protein levels [188]. This macronutrient configuration compels metabolic adaptation toward lipid oxidation and ketone body generation rather than glucose-dependent pathways. Beyond its established neurological applications [189], the regimen has attracted interest across diverse pathological contexts [190], though its therapeutic value remains highly condition-specific.

#### 7.5.1. Clinical Effectiveness

The intersection of ketogenic nutritional strategies with IBD presents a landscape marked by profound uncertainty and contradictory findings. Human evidence remains severely limited, consisting primarily of an uncontrolled observational report by Norwitz and Soto-Mota involving ten patients (six with UC, four with CD) who adopted carnivore-oriented ketogenic protocols [191]. This case series documented striking clinical improvements: complete symptomatic remission occurred across all participants, as did a decline in inflammatory markers. Quality of life assessments improved substantially, with mean IBD Questionnaire scores rising from 95 to 216 points. The authors proposed that a drastic reduction in fermentable carbohydrates and fiber fundamentally alters substrate availability for intestinal microorganisms, potentially suppressing disease-associated pathogenic species while promoting tolerogenic bacterial populations. Symptom recurrence followed carbohydrate reintroduction, suggesting dietary dependency. The intervention may function partly through elimination mechanisms, removing potential immunological triggers, including gluten, lectins, food additives, and fermentable oligosaccharides, disaccharides, monosaccharides, and polyols. Nevertheless, substantial safety and efficacy concerns persist [156].

#### 7.5.2. Mechanism of Action and Microbiome Effects

Experimental animal work shows the potential efficacy of this diet: investigations by Li and colleagues revealed detrimental outcomes, including heightened inflammation and compromised intestinal barrier function in murine colitis models [192], whereas Kong’s research team documented opposing results with diminished inflammatory infiltration and favorable shifts in microbial ecology [193]. The latter study specifically demonstrated the enrichment of *Akkermansia* populations alongside reductions in *Escherichia* and *Shigella* species. Following experimental colitis induction, ketogenic-fed animals maintained superior intestinal barrier integrity and exhibited decreased RORγt^+^ CD3^−^ group 3 innate lymphoid cells, accompanied by suppressed expression of proinflammatory mediators, including IL-17α, IL-18, IL-22, and Ccl4 [193]. These mechanistic effects appeared distinct from those produced by less restrictive low-carbohydrate approaches, suggesting unique metabolic consequences.

### 7.6. Low Emulsifier Diets

Dietary restriction of food-grade emulsifiers has gained attention as a potential therapeutic strategy following experimental animal research demonstrating their capacity to compromise intestinal barrier integrity and alter microbial ecosystems [194,195,196,197,198]. These additives, widely incorporated into manufactured foods for texture enhancement and preservation, have been shown in laboratory models to erode the mucosal protective layer, facilitate microbial translocation across the epithelium, and activate inflammatory cascades, with carboxymethylcellulose and polysorbate-80 being particularly implicated [194,195,196,197,198].

#### 7.6.1. Clinical Effectiveness

The current ECCO 2025 Consensus acknowledges that available published data remain inadequate to endorse emulsifier avoidance for achieving or sustaining remission in CD, although forthcoming complete results from the ADDapt trial may necessitate guideline revision [199]. Initial feasibility work by Sandall established that patients with CD can successfully implement emulsifier elimination and potentially experience symptomatic relief and improved disease management [200]. Subsequently, Fitzpatrick conducted an RCT comparing high versus low emulsifier consumption over four weeks, finding no measurable differences in disease activity indices between groups, though both dietary modifications yielded enhanced quality of life and reduced fatigue [201].

The ADDapt study represents a multicenter investigation utilizing a double-blind, placebo-controlled re-supplementation framework involving 154 participants with mild to moderate active CD [199]. This trial employed an innovative comparative approach wherein all participants initially followed a low-emulsifier diet, after which one cohort maintained emulsifier restriction while the control group received deliberate emulsifier reintroduction through blinded supplementation. Importantly, even the restriction group did not achieve complete emulsifier elimination, as some residual intake from dietary sources remained; the comparison therefore assessed the impact of relative reduction versus re-supplementation rather than absolute avoidance. Preliminary findings indicated superior clinical response rates and more pronounced fecal calprotectin reductions in the restriction group, suggesting that even partial emulsifier reduction—without complete elimination—may confer clinical benefit. This observation strengthens the hypothesis that these synthetic compounds contribute to disease pathogenesis through microbiome-dependent pathways, as meaningful clinical differences emerged despite imperfect dietary adherence.

#### 7.6.2. Mechanism of Action and Microbiome Effects

Laboratory evidence has documented that emulsifier exposure modifies microbial community structure, diminishes taxonomic diversity, enables bacterial penetration of the mucus barrier, and provokes subclinical inflammatory responses [195,196,197,202,203]. Human-derived tissue studies using ex vivo methodologies have similarly revealed alterations in both microbial populations and host gene expression patterns following emulsifier contact, though comprehensive characterization of microbiome transformations during emulsifier restriction in IBD patients awaits further investigation. Critically, the direct impact of low emulsifier diets on human gut microbiota composition and function in IBD remains undefined, representing a significant knowledge gap that future studies must address to elucidate the mechanistic basis of any observed clinical benefits.

### 7.7. Low-Sulfur Diets (4-SURE, UCED)

Dietary interventions targeting sulfur reduction have garnered attention in UC management, given the established connection between excessive hydrogen sulfide production and disease pathogenesis. When sulfur-containing dietary components undergo bacterial metabolism in the colon, they generate hydrogen sulfide, which at elevated concentrations can compromise the protective mucus barrier, interfere with the oxidative metabolism of colonocytes, and amplify inflammatory responses [204,205].

#### 7.7.1. Clinical Effectiveness

Current clinical practice guidelines from ECCO indicate that available evidence remains inadequate to support recommending either the 4-SURE diet or UC Exclusion Diet for achieving or maintaining disease remission in patients with UC [40]. The 4-SURE dietary framework, representing four distinct strategies for sulfide reduction, operates through multiple complementary pathways to optimize colonic health. Initial feasibility assessment involving twenty-eight individuals with mild to moderately active disease demonstrated excellent tolerability and adherence approaching 95%. Clinical response occurred in nearly half the participants, while endoscopic improvement was documented in approximately one-third. Laboratory parameters showed favorable trends, with FC declining from 400 to 175 micrograms per gram, fecal SCFAs increasing by 69%, and quality of life measures improving by ten points [206].

#### 7.7.2. Mechanism of Action and Microbiome Effects

Emerging evidence from mechanistic studies provides additional insight into how dietary sulfur reduction influences gut ecosystem dynamics and host metabolism. Mechanistic validation through deep functional profiling demonstrated that the 4-SURE diet achieved all of its intended microenvironmental targets [207]. Specifically, the diet significantly reduced the abundance of known hydrogen sulfide-producing taxa, including *Odoribacter* and *Peptostreptococcaceae*, altered expression of 12 sulfur-metabolizing genes, decreased the protein fermentation marker indole, and reduced ex vivo fecal H_2_S production by 45%. This approach simultaneously decreases consumption of total protein, sulfur-containing amino acids, and sulfur-based food additives while augmenting intake of resistant starch and non-starch polysaccharides. These modifications theoretically promote SCFA generation by beneficial microbiota and establish an acidic colonic milieu that inhibits the proliferation of sulfate-reducing bacterial species [206]. A pilot investigation employing shotgun metagenomic sequencing and untargeted metabolomics among individuals with mild-to-moderate UC demonstrated that reduced sulfur intake promoted significant alterations in both microbial community structure and metabolic profiles [208]. Participants adhering to sulfur restriction exhibited a decreased abundance of *Eggerthella lenta*, a pathobiont associated with intestinal dysbiosis and inflammatory processes, while simultaneously showing an expansion of beneficial microbial taxa, including *Collinsella stercoris*, *Asaccharobacter celatus*, and *Alistipes finegoldii* [208]. SCFA-producing bacteria, particularly *Faecalibacterium prausnitzii*, *Bacteroides ovatus*, *Parabacteroides distasonis*, *Blautia wexlerae*, and *Agathobaculum butyriciproducens*, demonstrated increased representation [208]. These microbial shifts corresponded with enhanced alpha diversity indices and meaningful reductions in lipopolysaccharide-binding protein concentrations, suggesting improved intestinal barrier integrity. Metabolomic analysis revealed upregulation of compounds involved in nitrogen metabolism, including uric acid, methyluric acid, and N-acetyl-L-glutamate, indicating enhanced nitrogen clearance mechanisms. Additionally, microbiome-derived metabolites such as indoleacetyl-glutamine and N-eicosapentaenoyl phenylalanine increased following sulfur restriction, potentially reflecting anti-inflammatory adaptations mediated through aryl hydrocarbon receptor activation pathways.

Taken together, these observations suggest that dietary modulation of sulfur metabolism and associated microbial communities may represent a promising avenue for therapeutic intervention, though rigorously designed RCTs with adequate sample sizes and appropriate control groups remain necessary to establish clinical efficacy and inform evidence-based practice recommendations.

### 7.8. Intermittent Fasting

Dietary fasting interventions have attracted growing attention in the management of IBD, with survey data indicating that roughly one-fifth to one-third of IBD patients incorporate various fasting protocols into their regimens [209]. These approaches encompass several distinct patterns: alternating between fasting and normal eating days, confining food consumption to specific daily time windows, and limiting caloric intake to two non-consecutive days weekly (the 5:2 protocol). The therapeutic interest derives from experimental observations suggesting that periods of caloric restriction may influence immune function, diminish inflammatory and oxidative processes, reshape the intestinal microbial ecosystem, and activate cellular self-renewal mechanisms [210].

#### 7.8.1. Clinical Effectiveness

An observational study from 2006 examining patients with UC during Ramadan documented improvements in clinical activity scores associated with the fasting period, whereas a subsequent prospective investigation reported elevations in Mayo scores following Ramadan observance, with this effect being more pronounced in elderly patients and those with higher baseline FC levels [211]. A retrospective comparison revealed no meaningful distinctions in disease activity between IBD patients who fasted and those who did not. In a landmark open-label RCT published in Nature Medicine, Kulkarni et al. randomized 97 adults with mild-to-moderate CD (CDAI 150–450) 2:1 to three monthly 5-day cycles of a fasting-mimicking diet (FMD; n = 65) or continuation of baseline diet (n = 32) [212]. The FMD is a plant-based, calorie-restricted diet (1090 kcal on day 1; 725 kcal on days 2–5, low in protein and sugar, high in unsaturated fat) consumed for only five consecutive days per month, with patients returning to their habitual diet for the remaining approximately 25 days. After three cycles, clinical response (defined as a CDAI decrease of at least 70 points or CDAI of 150 or less) was achieved in 69.2% of the FMD group versus 43.8% of controls (*p* = 0.03), and clinical remission (CDAI of 150 or less) was attained in 64.6% versus 37.5% (*p* = 0.02). Median CDAI decline was 105 points in the FMD group compared with 76 in controls (*p* = 0.02). Fecal calprotectin decreased by a mean of 22.0% in the FMD group compared with an increase of 8.0% in controls (*p* = 0.03), and a 50% or greater decline in fecal calprotectin was observed in 37.0% versus 6.3% of participants (*p* = 0.01). CRP showed a trend toward improvement in the FMD group but did not reach statistical significance (−1.0% vs. +36.9%, *p* = 0.06), possibly reflecting the predominantly mild disease severity in most enrolled patients. Subgroup analyses revealed that FMD was effective in both mild (75.0% vs. 47.8%, *p* = 0.03) and moderate (57.1% vs. 11.1%, *p* = 0.04) CD. Notably, clinical response was significantly higher in patients with colonic (82.4% vs. 33.3%, *p* = 0.01) and ileocolonic disease (71.4% vs. 30.0%, *p* = 0.03), but no benefit was observed in patients with isolated ileal disease (55.5% vs. 60.0%, *p* = 0.99). FMD was also effective in patients not receiving any medical therapy (76.9% vs. 33.3%, *p* = 0.04), an important finding given that there are currently no FDA-approved therapies for mild CD other than corticosteroids. Patient-reported outcomes similarly favored FMD, with PRO remission in 47.7% versus 25.0% (*p* < 0.05) and patient global assessment of remission in 24.6% versus 6.3% (*p* = 0.03) [212].

Dietary adherence was 76.9% across all three cycles, likely facilitated by the short duration of dietary restriction (only 5 days per month) and provision of commercial meal kits. FMD was well tolerated, with no severe adverse events reported in the intervention group; the most common side effects were mild fatigue (52.3%) and headache (50.8%), which are typical of caloric restriction. However, clinical benefits were not maintained after a 3-month washout period following the third cycle, suggesting that continued monthly FMD cycles may be necessary for sustained remission [212]. One recent pilot study of 32 patients with UC examined the effect of two 5-day intervals of a fasting-mimicking diet during the initiation of advanced therapy in an RCT, compared with a low-residue diet. After 8 weeks, there was no difference in clinical response to medications. However, clinical improvement (continuous numerical improvement in SCCAI), steroid tapering, and lower inflammatory markers (serum amyloid A protein) were found to be greatest in those in the intervention vs. control groups [213]. However, the 2025 ECCO guidelines conclude that existing data are inadequate to support intermittent fasting as a therapeutic strategy for achieving or sustaining remission in IBD, and larger, well-powered studies are needed [40].

#### 7.8.2. Mechanism of Action and Microbiome Effects

Experimental investigations using colitis models in rodents have yielded encouraging outcomes with fasting-mimicking dietary protocols. Research has shown that implementing four-day cycles of such diets reduced markers of intestinal inflammation while simultaneously expanding stem cell populations, promoting beneficial shifts in microbial communities, and protecting against DSS-induced intestinal injury in murine subjects [214]. Additional work demonstrated that three-day fasting-mimicking cycles decreased inflammatory markers and tissue damage, preserved colon length, and enhanced the proliferation of colonic crypts and stem cells [215]. When comparing different fasting methodologies, investigators found that both time-restricted feeding patterns and intermittent energy restriction offered protection against experimental colitis and associated behavioral manifestations, mediated through favorable alterations in gut microbial composition and preservation of the intestinal barrier. Critically, none of the existing human studies have incorporated comprehensive microbiome analyses, leaving the mechanistic basis of any clinical effects incompletely understood and limiting the ability to identify which patients might benefit most from fasting interventions.

The Kulkarni et al. trial provides the first human mechanistic data supporting the anti-inflammatory effects of FMD in CD. Untargeted plasma metabolomics revealed that 14 of the 20 most downregulated pathways after FMD mapped to arachidonic acid and linoleic acid metabolism, consistent with broad suppression of lipid mediator signaling [212]. Specifically, FMD significantly reduced downstream oxidation products of leukotriene B4 and leukotriene E4, pro-inflammatory mediators that are elevated in IBD and are known to drive neutrophil chemotaxis and epithelial barrier disruption. Oxidized linoleic acid derivatives (13-HODE, 9-HODE, and their downstream trihydroxyoctadecenoic acids), which are elevated in active IBD and correlated with calprotectin levels, were also significantly reduced after FMD. Conversely, FMD increased anti-inflammatory lipoxin metabolites (15-oxo-LXA4/LXB4), which suppress NF-κB-dependent transcription [212]. RT-qPCR analysis of PBMCs demonstrated that a single 5-day FMD cycle significantly reduced expression of TNF, NLRP3, IL-1β, IL-18, and the chemokines CCL20 and CXCL10, which mediate immune cell recruitment to sites of intestinal inflammation [212]. These concordant metabolomic and transcriptomic findings provide a molecular basis for the clinical improvements observed and suggest that FMD acts at the pathway level, attenuating both lipid-mediated and cytokine-mediated inflammatory cascades rather than targeting a single mediator. These results represent the first human evidence bridging the preclinical observation of FMD-mediated anti-inflammatory effects to clinical disease activity in IBD.

### 7.9. Other Dietary Interventions and Future Directions

Additional dietary approaches investigated in IBD include the McMaster Elimination Diet for CD (MED-CD), an IgG-based diet, milk-free diet, low carrageenan diet, and the Monash Pouch Diet (MPD). However, comprehensive microbiome analyses for many of these interventions remain limited.

Table 2 summarizes key findings from human studies of dietary interventions in IBD.

In synthesizing the current evidence base, dietary interventions clearly possess the capacity to substantially alter gut microbiome composition and metabolic activity in IBD patients, with these microbiological changes mechanistically linking to observed clinical benefits. However, no single dietary approach universally optimizes microbiome configuration across all patients, underscoring the need for individualized strategies considering disease phenotype, baseline microbial status, symptom patterns, and patient preferences. Future research efforts should prioritize establishing standardized methodologies for microbiome assessment in dietary intervention trials, identifying reliable microbiome-based biomarkers of treatment response, validating personalized nutrition algorithms through rigorous controlled trials, and elucidating the long-term effects of sustained dietary modifications on microbiome stability and disease trajectory. The ultimate goal remains developing evidence-based, microbiome-informed dietary recommendations that can be seamlessly integrated into comprehensive IBD management strategies to improve patient outcomes and quality of life.

## 8. Micronutrients

In patients with IBD, dietary supplementation with micronutrients is often recommended, and increasingly, the use of probiotics and prebiotics is also suggested. As reported in the ESPEN guidelines [67], patients with IBD are at risk of developing micronutrient deficiencies, particularly of iron, zinc, calcium, and vitamin D. During the active phase of the disease, serum levels of these micronutrients may be altered due to systemic inflammation, making their assessment during remission important for a more accurate evaluation of nutritional status.

A study conducted by Massironi et al. [216] highlighted that 78% of IBD patients in the early stages of the disease exhibited at least one micronutrient deficiency. These deficiencies can occur even in individuals who appear to be well-nourished. Therefore, monitoring serum levels of B vitamins, vitamin D, iron, and zinc is recommended in order to tailor supplementation interventions based on individual needs, thereby contributing to the maintenance of overall health and supporting clinical remission [20].

The levels of essential micronutrients can be influenced by several factors, including disease activity, dietary intake, supplement use, environmental exposure, and genetic variations that regulate the intestinal absorption, systemic transport, and metabolism of these elements. Moreover, the composition of the intestinal microbiota plays a crucial role in modulating the bioavailability and assimilation of nutrients. Analyzing interactions between genetic variants, including those associated with the microbiome, could help identify subgroups of patients with greater susceptibility to micronutrient deficiencies or an increased risk of developing related complications [217]

### 8.1. Role of Vitamins

#### 8.1.1. Vitamin D 

Numerous scientific studies indicate that vitamin D deficiency plays a key role in the pathogenesis of IBD [218,219]. Vitamin D is involved in various pathophysiological processes, including immune cell differentiation, modulation of the intestinal microbiota, regulation of gene transcription, and maintenance of intestinal epithelial barrier integrity [219]. It contributes to mucosal barrier homeostasis and plays a protective role against intestinal pathogens, including adherent-invasive *Escherichia coli* [220].

Furthermore, it has been hypothesized that vitamin D may have a prophylactic function against *Clostridium difficile infections*, to which IBD patients are particularly susceptible. This protective effect would be mediated by the induction of antimicrobial peptides, such as cathelicidins, and by the modulation of the intestinal microbiome composition [221]. Cathelicidins, in addition to having direct antimicrobial action, act as mediators of inflammation modulated by vitamin D in the context of infection.

#### 8.1.2. Vitamin B12

Recent studies based on Mendelian randomization approaches have examined the association between plasma levels of folate and vitamin B12 and the risk of developing autoimmune diseases, including IBD. In these studies, single-nucleotide polymorphisms (SNPs) that are significantly associated with circulating levels of folate and vitamin B12 were selected. The results indicated that higher levels of vitamin B12 may be associated with a slight increase in the risk of developing IBD (IVW: OR = 1.14, 95% CI: 1.03–1.26, *p* = 0.010; maximum likelihood: OR = 1.14, 95% CI: 1.01–1.29, *p* = 0.035; MR-PRESSO: OR = 1.14, 95% CI: 1.01–1.28, *p* = 0.037), but this association was not statistically significant after correction for multiple comparisons using Bonferroni’s method, suggesting that a random effect cannot be excluded [222]. The authors concluded that further studies are needed to clarify the potential causal role of vitamin B12 in the development of IBD.

In parallel, the study by Massironi et al. [216] found that approximately 22% of European IBD patients had a vitamin B12 deficiency, primarily secondary to ileal malabsorption due to surgical resection or inflammatory involvement of the ileal tract. This deficiency is associated with clinical manifestations such as macrocytic anemia and peripheral neuropathy. Therefore, vitamin B12 supplementation is recommended for patients who have undergone an ileal resection exceeding 20 cm or have active ileal disease [223].

From a biochemical perspective, vitamin B12 is an essential cofactor for two enzymes crucial in human metabolism: methionine synthase, involved in homocysteine metabolism, and methylmalonyl-CoA mutase, essential for the catabolism of odd-chain fatty acids and certain amino acids [224]. Both processes are vital for the proper functioning of the nervous system and for cellular energy production.

Vitamin B12 is synthesized exclusively by bacteria through the transformation of compounds found in foods of animal origin. However, the role of vitamin B12 in modulating the composition and function of the intestinal microbiota remains underexplored. A recent systematic review suggested possible effects of vitamin B12 on the alpha and beta diversity of the intestinal microbiota and on the production of SCFAs, although the available results are not yet conclusive [224].

### 8.2. Role of Minerals

Iron, calcium, and zinc are the micronutrients most frequently associated with inadequate dietary intake in patients affected by IBD. In the United Kingdom, 42% of IBD patients with a disease duration of less than 18 months exhibit iron deficiency [20]. Globally, it is estimated that 10–13% of the population shows calcium deficiency [225,226]; however, the assessment of serum calcium in IBD patients is not considered a reliable indicator of mineral status, making bone Dual-Energy X-ray Absorptiometry (DEXA) necessary for an accurate estimation of calcium body stores. Zinc also shows a global deficiency prevalence ranging from 40% to 50% [225], highlighting the importance of regular monitoring of serum levels and suggesting empirical supplementation in patients with persistent chronic diarrhea.

#### 8.2.1. Iron

Iron deficiency and its supplementation can significantly impact the composition of the gut microbiota. In particular, oral iron supplementation, if not properly formulated, may induce oxidative stress and damage the intestinal epithelium. Recent studies using shotgun metagenomic sequencing in animal models [227] have challenged previous evidence, demonstrating that iron deficiency in conditions of iron deficiency anemia is associated with profound alterations in colonic microbial communities, including an increase in species belonging to the genus Clostridium and a higher production of SCFAs, both simple and branched. In parallel, colonic epithelial deterioration has been observed, with changes in gene and protein expression of extracellular matrix components involved in intestinal barrier functionality. Additionally, elevated levels of blood lipopolysaccharides (LPS) and an intensified immune response to dysbiotic bacteria have been reported [228]. Previous studies had already documented alterations in fecal SCFA content related to iron concentration, reporting reduced levels of butyrate and propionate during iron deficiency states in rats [229]. The relationship between gut microbiota and iron metabolism is bidirectional. On one hand, microbiota composition influences iron absorption: studies in germ-free or antibiotic-treated animals demonstrate that microbiota absence reduces systemic iron absorption and retention by up to 25%. On the other hand, oral iron supplementation can adversely affect gut microbiota composition, promoting the expansion of potentially pathogenic Enterobacteriaceae while reducing beneficial *Bifidobacteria* and *Lactobacilli*, which may exacerbate intestinal inflammation in IBD patients [21].

#### 8.2.2. Zinc

Zinc (Zn^2+^) deficiency is also frequently observed in IBD patients [230], where alterations in the expression of specific transporters and ion channels are noted. Zinc deficiency is associated with increased intestinal permeability and an overall decline in gastrointestinal health [231]. Zinc is an essential trace element, crucial for maintaining redox homeostasis and intestinal microbiota stability [232]. Since the human body lacks significant tissue reserves of zinc, a constant dietary intake is necessary. Plant-based or high-carbohydrate diets, especially in physically active individuals or patients with high nutritional demands, such as those with IBD, should be carefully balanced and, if necessary, supported by fortified foods [233]. In vitro studies have shown that the bioavailability of zinc present in plants, such as lettuce, largely depends on the presence of the colonic microbiota, which utilizes unabsorbed zinc from the proximal gastrointestinal tract, making it accessible to the host. In inflammatory conditions, intestinal pathogens compete with commensal bacteria for zinc, further reducing its bioavailability [234]. However, excessive dietary zinc can also have negative effects: murine model studies have demonstrated reduced resistance to *Clostridioides difficile* infection in the presence of high dietary zinc levels [235].

#### 8.2.3. Calcium

Calcium is the most abundant mineral in the human body, accounting for approximately 1.5–2% of total body weight. IBD patients are particularly at risk of developing hypocalcemia, osteopenia, and osteoporosis [234,236], due to: (i) intestinal malabsorption secondary to dysfunction of Ca^2+^ transporters [237,238]; (ii) dietary restrictions, particularly the exclusion of dairy products [239]; (iii) prolonged use of corticosteroids, commonly employed in IBD management [240]. Calcium can also influence gut microbiota composition through lumen pH modulation and by exerting preservative and buffering effects [241]. Calcium supplementation has been associated with the promotion of the growth of Bifidobacterium and Bacteroides [242], which produce lactate and propionate, respectively, fueling butyrate production through cross-feeding mechanisms by other bacterial species [13]. Additionally, it is known that Bifidobacterium modulates the activity of specific inflammatory cytokines, including IL-8 and IL-12. In cases of inadequate dietary intake, calcium is mobilized from the skeleton, increasing the risk of osteoporosis; therefore, proper supplementation should be considered in IBD patients.

## 9. Drug–Nutrient Interactions

Pharmacological management of IBDs, while essential for disease control, significantly impacts micronutrient status through mechanisms including altered absorption, increased metabolic demands, and direct interference with nutrient metabolism (Table 3). Understanding these drug–nutrient interactions is crucial for preventing deficiencies that compromise treatment outcomes and patient quality of life.

### 9.1. Corticosteroids

Systemic corticosteroids, frequently employed during acute IBD flares, exert profound effects on bone and mineral metabolism through multiple mechanisms: decreased intestinal calcium absorption via antagonism of vitamin D action at the enterocyte level, increased urinary calcium excretion, inhibition of osteoblast-mediated bone formation, enhanced osteoclastic bone resorption, and impaired skeletal muscle function [243,244,245]. Steroids decrease calcium absorption from the intestine and increase urinary calcium loss, leading to secondary hyperparathyroidism and increased bone resorption [246]. Additionally, corticosteroids directly inhibit osteoblast function and promote osteoblast apoptosis while extending osteoclast lifespan, resulting in an imbalance favoring bone resorption over formation [247]. Rapid bone mineral density loss is most pronounced during the initial three to six months of therapy, with bone mineral density potentially decreasing by 10–20% in the first year of treatment [248,249].

Beyond calcium and vitamin D, corticosteroids can affect potassium and magnesium homeostasis, leading to urinary losses of these electrolytes [250,251]. Potassium and magnesium depletion can result in muscle weakness, cardiac arrhythmias, and neurological symptoms. Protracted corticosteroid therapy, combined with the inflammatory cytokines that activate NF-κB and lower anabolic hormones, contributes to protein catabolism and muscle wasting, characteristic of sarcopenia, in IBD patients [250,251].

It is recommended that patients with IBD receiving systemic corticosteroids have an adequate intake of calcium (1000–1200 mg daily) and vitamin D (600–800 IU daily, though higher doses may be needed to achieve target 25-hydroxyvitamin D levels greater than 50 nmol/L or 30 ng/dL) to mitigate bone loss [40,67]. It is important to note that topically acting corticosteroids with low systemic bioavailability, such as budesonide (approximately 10–15% systemic absorption) and beclomethasone dipropionate (approximately 10–15% systemic absorption), have substantially lower effects on bone metabolism compared to systemic corticosteroids due to extensive first-pass hepatic metabolism [252,253]. However, supplementation strategies must account for disease-related malabsorption, particularly in patients with active inflammation or extensive ileal involvement, who may require higher doses or alternative administration routes [254]. Associations with dietary approaches require particular attention. While the MD and other anti-inflammatory dietary patterns naturally provide calcium-rich foods and vitamin D sources, dietary measures alone are insufficient to counteract corticosteroid-induced depletion; supplementation remains necessary but should complement dietary optimization [255]. Patients following plant-based or vegan diets require particular attention to ensure adequate calcium and vitamin D intake from fortified products and supplements, as these restrictive patterns may compound the risk of deficiency [256]. Additionally, patients with limited sunlight exposure due to photosensitivity, geographic location, or lifestyle factors may require higher vitamin D supplementation doses to maintain adequate status [257]. Older adults have lower vitamin D levels due to reduced skin synthesis and decreased dietary intake, making adequate supplementation particularly important in elderly IBD patients receiving corticosteroids [258].

### 9.2. Aminosalicylates

Sulfasalazine, an aminosalicylate compound consisting of 5-aminosalicylic acid linked to sulfapyridine that exerts anti-inflammatory effects in the intestinal mucosa, impairs folate absorption through direct interference with intestinal folate transporters by competitive inhibition of the reduced folate carrier and folate hydrolase at the intestinal brush border, thereby preventing the conversion of dietary polyglutamate folates to the absorbable monoglutamate form [259,260]. This dose-dependent effect creates clinically significant deficiency risk, compounded by baseline increased folate requirements in IBD due to accelerated cell turnover in inflamed mucosa, chronic blood loss, and potential dietary restrictions [223]. The prevalence of folate deficiency in patients with IBD receiving sulfasalazine can reach 28–30%, significantly higher than in untreated populations [223,261].

The implications of folate deficiency extend beyond macrocytic anemia to include glossitis, stomatitis, and fatigue. In IBD populations, inadequate folate status has been associated with elevated colorectal cancer risk, with meta-analyses suggesting that folic acid supplementation may reduce colorectal cancer risk by approximately 40% [262,263]. Furthermore, women of childbearing age with IBD require high-dose folic acid supplementation (5 mg daily) initiated at least one month before conception and continued through the first trimester to reduce neural tube defect risk by up to 70% [264,265].

Prophylactic folic acid supplementation is recommended for all patients receiving sulfasalazine. According to current ECCO guidelines, serum folate should be monitored at diagnosis and annually thereafter in all IBD patients, with particular attention to those on restrictive diets, those receiving sulfasalazine or methotrexate, and women planning pregnancy [40]. Comparative studies demonstrate that both folic acid and folinic acid effectively restore body folate stores, though folinic acid exhibits superior bioavailability and may be more effective in severe deficiency states [266]. Standard supplementation with folic acid 1 mg daily is typically sufficient for most patients, with monitoring of folate levels every three to six months advisable when additional risk factors exist [67]. Dietary counseling should emphasize folate-rich foods, including leafy green vegetables, legumes, fortified cereals and grains, citrus fruits, and liver. However, given the mechanism of sulfasalazine-induced malabsorption at the intestinal transporter level, dietary folate alone cannot overcome the drug effect, necessitating pharmacological supplementation. Furthermore, women of childbearing age with IBD require folic acid supplementation initiated at least one month before conception and continued through the first trimester to reduce neural tube defect risk by up to 70% [40,267]. For women receiving sulfasalazine, 2 mg folic acid daily for 3 months prior to conception is recommended. For women with extensive small bowel disease, malabsorption, or a family history of neural tube defects, 5 mg folic acid daily is recommended.

### 9.3. Methotrexate

Methotrexate functions as a folate antagonist by inhibiting dihydrofolate reductase, the enzyme that catalyzes the reduction of dihydrofolic acid to tetrahydrofolic acid, thereby depleting the tetrahydrofolate cofactors required for the one-carbon transfer reactions essential for DNA synthesis, amino acid metabolism, and cellular proliferation [268]. This mechanism underlies both its therapeutic immunosuppressive effects and its adverse event profile, which includes myelosuppression, hepatotoxicity, mucositis, and gastrointestinal symptoms [269].

Folate supplementation has been demonstrated to partially mitigate methotrexate toxicity without compromising therapeutic efficacy when appropriately timed. The protective effect of folate supplementation reduces the risk of gastrointestinal adverse events by approximately 26%, liver enzyme elevations by 76%, and overall discontinuation due to adverse events by 61% [269]. Current guidelines recommend folic acid supplementation in all IBD patients receiving methotrexate: either 5 mg once weekly administered 24–72 h after methotrexate dosing, or 1 mg daily for five days per week, excluding the day of methotrexate administration [67]. The timing is crucial to reduce adverse effects (glossitis, stomatitis, nausea, hepatotoxicity, myelosuppression) while avoiding interference with methotrexate’s therapeutic action by ensuring the drug has sufficient time to exert its inhibitory effects before folate restoration [270].

Women of childbearing potential require counseling regarding adequate folate supplementation during methotrexate therapy, though methotrexate itself is highly teratogenic and strictly contraindicated in pregnancy, requiring discontinuation at least 1 to 3 months (ideally 1 month minimum) before conception attempts to allow for clearance of methotrexate conjugates and transition to other appropriate therapies [267,271]. Folic acid consumption in the presence of dihydrofolate reductase inhibitors (including methotrexate) reduces the relative risk of developing cardiac defects, and as such, folic acid supplementation should be continued or initiated to reduce the risk of adverse effects if accidental exposure occurs [267]. Men receiving methotrexate should also discontinue the medication at least 1 to 3 months before attempting conception due to potential effects on spermatogenesis. Beyond folate, methotrexate may affect vitamin B12 and vitamin B6 status, can interfere with B12 absorption through effects on the ileum, and may increase homocysteine levels [272]. Patients on methotrexate should undergo comprehensive micronutrient screening at baseline and periodically during treatment, typically every 3–6 months [67].

### 9.4. Thiopurines

Azathioprine and 6-mercaptopurine, while not directly causing micronutrient malabsorption through effects on intestinal absorption, induce macrocytosis through myelosuppressive activity via incorporation of thioguanine nucleotides into DNA rather than true folate or vitamin B12 deficiency [67,273]. This distinction is clinically relevant: the macrocytosis observed with thiopurines, characterized by mean corpuscular volume elevation, typically in the range of 100–110 fL, does not typically require vitamin supplementation unless concurrent true deficiencies exist from IBD-related malabsorption or dietary inadequacy [274].

The primary nutritional consideration with thiopurines involves supporting adequate hematopoiesis in the context of bone marrow suppression. Thiopurines can cause leukopenia, thrombocytopenia, and anemia through dose-dependent myelosuppression, with serious toxicity occurring in 9–25% of patients [275]. Patients require regular complete blood count monitoring, with frequency determined by treatment phase and individual risk factors, including thiopurine methyltransferase enzyme activity [276,277,278,279]. While thiopurines do not mandate routine prophylactic micronutrient supplementation beyond standard IBD screening, patients should maintain adequate intake of nutrients supporting hematopoiesis: iron, vitamin B12, folate, and protein [67]. Women with IBD who are pregnant or attempting conception may continue maintenance thiopurine therapy, as data do not demonstrate an increased risk of congenital malformations or infant infections, with no supplement requirement being necessary for all patients [267].

### 9.5. Biologic Therapies

Biologic agents, including anti-TNF antibodies (infliximab, adalimumab, golimumab, certolizumab), anti-integrin therapy (vedolizumab), anti-IL12/23 agents (ustekinumab), and anti-IL23 (risankizumab, mirikizumab, guselkumab), are not known to directly cause micronutrient depletions through pharmacological mechanisms affecting absorption or metabolism. However, the relationship between biologics and nutritional status is bidirectional and clinically significant.

Malnutrition negatively impacts biologic therapy outcomes through multiple mechanisms. Reduced serum albumin concentrations (typically <35 g/L) and protein-energy malnutrition correlate with increased inflammatory burden, accelerated infliximab clearance, diminished mucosal healing rates, and reduced clinical response to anti-TNF therapy [280,281]. The pharmacokinetic implications are substantial, as biologics, being large protein molecules, depend on adequate protein stores and albumin concentrations for optimal distribution and half-life. In a large population-based cohort study, protein-energy malnutrition was independently associated with earlier treatment failure in patients with CD, with a fifth of patients receiving only one dose due to early discontinuation [216]. Nutritional stats should be addressed prior to and during biologic therapy.

Sarcopenia, prevalent in 40–60% of IBD patients regardless of body mass index, associates with poorer biologic response through an odds ratio of 2.93 for primary non-response to anti-TNF treatment, higher treatment discontinuation rates, and increased postoperative complications [282,283,284]. The mechanisms linking sarcopenia to poor biologic response likely involve altered drug pharmacokinetics, persistent systemic inflammation driven by muscle-derived inflammatory mediators, impaired tissue repair capacity, and reduced functional reserve to tolerate disease and treatment-related stresses [285,286].

Obesity similarly affects biologic efficacy, though through distinct pathways. Patients with obesity demonstrate worse responses to biologics due to altered pharmacokinetics, including increased volume of distribution and accelerated drug clearance, combined with chronic low-grade inflammation mediated by adipose tissue-derived cytokines that create a proinflammatory milieu, reducing therapeutic efficacy [287]. Weight-based dosing strategies and higher maintenance doses may be necessary in obese patients, though optimal approaches remain under investigation [288].

Conversely, successful biologic therapy that achieves mucosal healing improves nutrient absorption capacity, potentially reversing disease-related micronutrient deficiencies that result from chronic inflammation, malabsorption, and increased intestinal losses [289]. The nutritional impact of biologics operates through several mechanisms. Anti-TNF therapy directly improves nutritional status by halting catabolic processes mediated by TNF-α, which causes cachexia through effects on leptin regulation, adipose tissue metabolism, and muscle protein breakdown [290,291]. Prospective studies in patients with active CD have demonstrated that infliximab therapy improves nutritional parameters, including body weight, body mass index, serum albumin, and lean body mass, with effects most pronounced in patients with baseline malnutrition and small bowel disease location [292]. The mechanism appears to involve both a reduction in intestinal inflammation, leading to improved absorption, and direct effects on metabolic pathways regulating appetite and body composition [293]. Nutritional indicators, including the Nutritional Risk Index, Malnutrition Universal Screening Tool, and Controlling Nutritional Status score, have been shown to predict clinical remission at week 14 following vedolizumab therapy in patients with UC, suggesting that baseline nutritional status influences therapeutic response across biologic classes [294]. However, no evidence currently suggests that any particular biologic agent is preferable to others from a nutritional perspective, or that biologics are more effective than other immunosuppressive therapies (such as thiopurines) in improving nutritional status. Notably, corticosteroids, while effective for inducing remission, have well-documented adverse effects on nutritional status, including protein catabolism, impaired calcium and vitamin D metabolism, and glucose intolerance, making them less favorable from a nutritional perspective compared to both biologics and other steroid-sparing therapies. For other biologic classes, emerging evidence suggests similar benefits. While data for vedolizumab, ustekinumab, and IL-23 inhibitors remain more limited, the general principle that effective suppression of inflammation through any mechanism should improve nutritional status appears to hold.

Recent advances in microbiome research have revealed an additional dimension to the nutrition–biologic therapy relationship. The gut microbiome, which is profoundly influenced by dietary intake and nutritional status, appears to predict therapeutic response to biologic agents. In a prospective study of patients initiating vedolizumab therapy, baseline gut microbiome composition and function significantly predicted clinical remission, with higher community diversity and enrichment of specific butyrate-producing bacteria, including *Roseburia inulinivorans,* associated with favorable outcomes [295]. Importantly, microbial metabolic pathways, including branched-chain amino acid synthesis and butyrate production pathways, were significantly enriched in baseline samples from patients with CD achieving remission, suggesting that nutritional substrates supporting beneficial microbial metabolism may influence therapeutic efficacy [296]. These findings provide mechanistic insights linking dietary interventions, which shape microbiome composition and function, to biologic treatment outcomes. The production of SCFAs, particularly butyrate, depends on adequate dietary fiber intake and appears to create a mucosal environment more conducive to therapeutic response.

The synergistic potential of combining biologics with nutritional interventions represents an evolving therapeutic strategy with substantial promise. Studies evaluating concomitant partial enteral nutrition with infliximab demonstrated superior maintenance of remission compared to infliximab alone (34% vs. 64% relapse rate at one year), supporting the concept of integrating pharmacological and nutritional approaches [297,298]. The mechanisms underlying this synergy likely involve nutritional support reducing inflammatory burden by providing adequate substrate for mucosal repair, modulating the gut microbiome favorably, reducing oxidative stress through provision of antioxidant micronutrients, and enhancing biologic drug efficacy by improving albumin levels and overall pharmacokinetic parameters [299,300].

### 9.6. Small Molecules

Janus kinase inhibitors, including filgotinib and tofacitinib (approved for UC) and upadacitinib (approved for both UC and CD), represent a newer therapeutic class targeting intracellular signaling pathways involved in immune and inflammatory responses [301,302]. Current evidence does not indicate direct micronutrient depletion effects with JAK inhibitors through mechanisms affecting absorption or metabolism. However, these agents require laboratory monitoring, including complete blood counts and lipid profiles, as they may cause anemia, neutropenia, lymphopenia, and lipid alterations, including increases in total cholesterol, LDL cholesterol, and HDL cholesterol [303,304]. While these hematologic effects do not represent direct micronutrient depletions, they necessitate adequate nutritional support for hematopoiesis, including ensuring adequate iron, folate, vitamin B12, and protein status [305]. The lipid effects warrant attention to cardiovascular risk management through dietary and, if necessary, pharmacological interventions [306]. Given the lipid effects of JAK inhibitors, dietary interventions emphasizing heart-healthy fats become particularly relevant. The MD, with its cardioprotective properties, may help manage metabolic parameters while providing anti-inflammatory benefits complementary to JAK inhibitor mechanisms [111].

Sphingosine-1-phosphate receptor modulators (ozanimod, etrasimod) approved for UC similarly lack evidence for direct micronutrient depletion, though long-term nutritional data are emerging [307,308,309,310]. These agents do not appear to significantly affect nutrient absorption or metabolism based on current understanding, though continued surveillance for unexpected nutritional effects remains important as clinical experience accumulates.

## 10. Preoperative Nutritional Optimization

Malnutrition represents a prevalent concern in patients with IBD scheduled for surgical intervention. The reported worldwide prevalence of protein-energy malnutrition ranges from 6.1% to 92.5%, with this wide variability likely attributable to heterogeneous definitions and assessment methods [216]. In a systematic review, 42% of patients with IBD were diagnosed with sarcopenia [216], and pooled adjusted data from a meta-analysis demonstrated that sarcopenic patients had higher odds of requiring surgery (adjusted OR 2.665; 95% CI 1.121–6.336; *p* = 0.027) and postoperative complications (adjusted OR 6.097; 95% CI 1.756–21.175; *p* = 0.004) [311]. Preoperative malnutrition is associated with surgical site infections, anastomotic leaks, delayed wound healing, prolonged hospital stays, and increased healthcare costs [312]. It has been reported that 47% of patients with CD and 16% of patients with UC undergo at least one surgical procedure during their lifetime [313].

Current guidelines recommend a systematic nutritional assessment before planned surgery. The ESPEN working group defines severe nutritional metabolic risk as the presence of at least one of the following: weight loss exceeding 10–15% within six months, Body Mass Index (BMI) below 18.5 kg/m^2^, Nutritional Risk Screening score greater than 5, or serum albumin below 30 g/L in the absence of hepatic or renal dysfunction [67]. Multiple guidelines recommend screening IBD patients preoperatively and initiating nutritional therapy when malnutrition is detected, even if surgery must be delayed [40,67,109].

Exclusive enteral nutrition (EEN) has emerged as a particularly effective preoperative optimization strategy. In the largest meta-analysis to date, Krasnovsky et al. pooled data from 14 studies comprising 874 EEN-treated and 1044 control patients and demonstrated that preoperative EEN was associated with a 2.1-fold reduction in intra-abdominal septic complications (RR 0.47; 95% CI 0.35–0.63; I^2^ = 0.0%) [314]. A 1.6-fold reduction in skin and soft tissue infections was also observed (RR 0.63; 95% CI 0.42–0.94; I^2^ = 42.7%) [314]. Notably, EEN significantly improved serum albumin (Standardized mean difference [SMD] 0.55; 95% CI 0.34–0.77) and reduced C-reactive protein levels (SMD −0.87; 95% CI −1.21 to −0.53) [6]. In contrast, total parenteral nutrition showed no significant benefit for infectious outcomes [314].

The 2025 ECCO Consensus recommends the provision of preoperative nutrition for patients with CD awaiting surgical intervention, with most evidence supporting four weeks of EEN [40]. A minimum of 10 days of oral nutritional supplementation may be considered if EEN is not feasible. Preoperative EEN for at least two weeks can reduce inflammatory burden and potentially prevent planned surgery; one case–control study reported that 25% (13/51) of participants treated with preoperative EEN avoided surgery entirely, compared with all participants proceeding to surgery in the control group [315].

In a prospective study by Costa-Santos et al., preoperative EEN (median duration 41.5 days) in malnourished adults with complicated CD resulted in significant improvements in disease activity (Harvey-Bradshaw Index: 8.7 ± 1.9 vs. 4.1 ± 2.4; *p* = 0.001), C-reactive protein (11.7 ± 10.3 vs. 0.8 ± 0.8 mg/dL; *p* = 0.008), and serum albumin (3.1 ± 0.6 vs. 4.0 ± 0.6 g/dL; *p* = 0.022) [7]. Notably, 20% of patients no longer required surgery after EEN, and despite being malnourished at baseline, those treated with EEN had postoperative complication rates comparable to well-nourished patients [109,316]. EEN tolerance was 83% in this cohort. A systematic review by Rocha et al. confirmed that EEN was well tolerated and served as an independent protective factor against infectious and noninfectious complications [109].

Emerging evidence also supports the CDED in the preoperative setting. Wall et al. conducted the first randomized controlled trial comparing six weeks of preoperative EEN, CDED with partial enteral nutrition, or standard care in adults with CD undergoing elective surgery [317]. CDED was well tolerated, with high adherence (80% of patients achieving >90% compliance), whereas EEN was less well tolerated, with 67% of patients withdrawing or changing treatment. CDED, which includes moderate amounts of fiber from whole, peeled fruits and vegetables, did not exacerbate gastrointestinal symptoms and may represent an alternative preoperative optimization strategy when EEN adherence is challenging.

Parenteral nutrition (PN) is reserved for specific clinical scenarios. Short-term PN may be considered in patients with intra-abdominal abscesses or phlegmonous inflammation limiting enteral access, high-output gastrointestinal fistula, prolonged ileus, or severe malnutrition when enteral nutrition has failed [109]. The enteral route should be preferred except when contraindicated by intestinal obstruction, ileus, severe shock, intestinal ischemia, high-output fistula, or severe intestinal hemorrhage [67].

When malnutrition is diagnosed, ESPEN guidelines recommend delaying IBD surgery for 7–14 days whenever possible to allow intensive nutritional therapy; this timeframe may be longer in patients with IBD [67,102]. Enhanced recovery after surgery (ERAS) principles should be adopted, including avoidance of prolonged preoperative fasting and early re-establishment of oral feeding postoperatively [67,318]. Early postoperative nutrition is associated with significant reductions in total complications and does not impair anastomotic healing [319].

The impact of preoperative nutritional optimization on gut microbiota composition represents an emerging area of investigation. Costa-Santos et al. demonstrated that EEN significantly altered overall microbial composition (PERMANOVA *p* = 0.046) and reduced α diversity (8 ± 2.3 vs. 5.2 ± 1.5; *p* = 0.023), primarily through decreased Enterobacteriaceae [316]. This reduction in gut microbial diversity in response to a chemically defined diet may represent a mechanism of action of EEN [78,316]. Notably, α diversity returned to near-baseline values within six months after surgery, suggesting that these microbiota changes are not long-lasting.

## 11. Knowledge Gaps and Limitations

Despite substantial progress, several critical gaps remain. The heterogeneity of IBD phenotypes, combined with individual variation in baseline microbiota composition, genetic background, and dietary habits, means that no single dietary intervention is universally effective. Current evidence is strongest for EEN and CDED in pediatric CD and for the Mediterranean diet as a general anti-inflammatory framework, yet large-scale randomized controlled trials with standardized microbiome endpoints are lacking for most other dietary approaches [320]. The emerging mechanistic understanding of how diet modulates gut microbiota function through SCFA production, bile acid metabolism, and tryptophan signaling pathways provides a rational basis for precision nutrition strategies, but translating this knowledge into clinical practice requires validated biomarkers that can predict individual dietary responsiveness [321]. Indeed, a recent longitudinal study employing shotgun metagenomics and causal mediation analysis in 198 adults (100 controls, 49 CD, 49 UC) demonstrated that habitual diet influences intestinal inflammation through fundamentally distinct microbiome pathways depending on disease subtype: in CD, specific bacterial taxa and metabolites mediated the anti-inflammatory effects of coffee, whole wheat bread, and healthier dietary patterns on the Harvey-Bradshaw Index, whereas in UC, Mediterranean-like diets and fruit intake reduced CRP through broader effects on microbial richness, reduced dysbiosis, and SCFA-related functional pathways [322]. These findings provide empirical support for the development of disease-specific, microbiome-informed dietary recommendations rather than uniform dietary advice across IBD subtypes.

From a practical standpoint, shared decision-making has been identified as a key strategy to improve patient adherence to dietary interventions. Engaging patients in conversations about available dietary options, with the advantages and limitations of each approach clearly explained, allows dietitians to facilitate informed treatment choices that reflect individual preferences and circumstances [321]. We believe that the integration of multi-omics technologies (metagenomics, metabolomics, proteomics) with longitudinal clinical data will be essential for developing evidence-based, personalized dietary recommendations. Furthermore, the field would benefit from harmonized study designs, including standardized dietary assessment tools, consistent microbiome analytical pipelines, and clinically meaningful composite endpoints that capture both symptomatic and objective measures of disease activity.

This review has several limitations that should be acknowledged. As a narrative review, it does not follow the systematic methodology of a systematic review or meta-analysis, and therefore, the literature selection may be subject to potential bias. The rapidly evolving nature of microbiome research means that some findings cited here may be superseded by newer studies. Additionally, the heterogeneity of study designs, populations, dietary interventions, and microbiome analytical methods across the reviewed literature makes direct comparisons between studies challenging. Many of the dietary intervention studies reviewed were conducted in specific populations (e.g., pediatric, Western cohorts) and may not be generalizable to all IBD patients globally. The microbiome data discussed are predominantly based on 16S rRNA gene sequencing or shotgun metagenomics, which provide compositional information but may not fully capture the functional metabolic activity of the gut microbiota. Finally, while we have attempted to critically appraise conflicting evidence where it exists, the narrative format inherently limits the quantitative synthesis of effect sizes across studies.

## 12. Conclusions

Diet and intestinal microbiome represent critical bidirectional therapeutic targets in IBD management, with accumulating evidence demonstrating that specific dietary interventions can induce remission, modulate inflammation, and reshape gut microbial communities toward health-associated profiles enriched in butyrate-producing bacteria while reducing pathobionts. Future IBD care should integrate personalized, microbiome-informed dietary strategies as complementary components of comprehensive treatment plans, requiring multidisciplinary collaboration, rigorous clinical trials with standardized microbiome analyses, and precision nutrition algorithms that account for disease phenotype, baseline microbial composition, and individual patient characteristics to optimize therapeutic outcomes.

Critically, achieving this integration depends on the availability of dietitians with IBD-specific expertise who can marshal evolving evidence, personalize dietary interventions, and coordinate them with pharmacological and surgical therapy within the multidisciplinary team [321].

## Figures and Tables

**Table 1:** Mapping of recommendations for dietary interventions in patients with inflammatory bowel disease, adapted from current clinical practice guidelines [40].

Diet	CD Induction	CD Maintenance	UC Induction	UC Maintenance	IBS-like	Other
EEN	✓					
CDED + PEN	✓					
PEN	✓	✓				
Tasty and Healthy	✓					
CD-TREAT	✓					
Curcumin/QingDai			✓	✓		
Mediterranean Diet				✓		
Red meat reduction				✓		
Low-FODMAP					✓	
SCD						✓
High/Low Fiber						✓
Gluten-Free						✓
Ketogenic						✓
IBD-AID, AIP						✓
Plant-Based						✓
Low-Emulsifier						✓
Low-Sulfur (4-SURE, UCED)						✓
Intermittent Fasting						✓

**Table 2:** Main gut microbiome alterations found in human studies on dietary interventions of patients with Inflammatory Bowel Disease. Within each box, ↑ denotes increased and ↓ denotes decreased relative abundance of the listed bacterial taxa following the respective dietary intervention.

Diet	Disease	Increased Bacteria	Decreased Bacteria
Mediterranean Diet	CD	↑ *Bacteroidetes*, *Clostridium clusters*	↓ *Proteobacteria*, *Bacillaceae*
	UC	↑ *Faecalibacterium prausnitzii*, *Dorea longicatena*, *Roseburia inulinivorans*, *Ruminococcus*, *Flavonifractor*, *Clostridium*, *Lactococcus*, *Blautia A*	↓ *Bifidobacterium*, *Blautia*, *Veillonella*, *Streptococcus*, *Massilioclostridium*
	IBD (mixed)	↑ *Roseburia*, *Lachnospira*, *Prevotella*, *Ruminococcus*, *Flavonifractor*, *Clostridium*, *Lactococcus*, *Blautia*	↓ *Ruminococcus torques*, *Ruminococcus gnavus*
Low-FODMAP	IBD (remission)	↑ *Bifidobacterium longum*, *Ruminococcus torques*	↓ *Faecalibacterium prausnitzii*, *Bifidobacterium adolescentis*, *Bifidobacterium dentium*, *Clostridium cluster XIVa*, *Akkermansia muciniphila*
	CD (remission)	↑ *Clostridium cluster XIVa*, *Akkermansia muciniphila*	
Low-Fat Diet	UC	↑ *Bacteroidetes*, *Prevotella*, *Faecalibacterium prausnitzii*	↓ *Actinobacteria*
Anti-Inflammatory Diet (IBD-AID)	CD	↑ *Roseburia hominis*, *Clostridia class*, *Eubacterium eligens*	↓ *Parabacteroides distasonis*
	UC	↑ *Faecalibacterium prausnitzii*, *Eubacterium eligens*, *Coprococcus catus*, *Bacteroides species*	↓ *Parabacteroides distasonis*
Specific Carbohydrate Diet	IBD (mixed)	↑ *Roseburia*, *Lachnospiraceae*, *Blautia*, *Faecalibacterium prausnitzii*, *Anaerobutyricum*, *Eubacterium eligens*, *Clostridium species*	
	CD (pediatric)	↑ *Eubacterium*, *Ruminococcus*, *Subdoligranulum*	↓ *Proteobacteria*, *Bacteroides*, *Parabacteroides*
	CD (adults)	↑ *Enterobacteriaceae*	↓ *Faecalibacterium prausnitzii*, *Eubacterium eligens*, *E. rectale*
Exclusive Enteral Nutrition	CD (pediatric)	↑ *Bacteroidetes*, *Alistipes*	↓ *Firmicutes*, *Faecalibacterium*, *Proteobacteria*
	CD (responders)	↑ *Faecalibacterium*, *Roseburia*	
CDED + Partial EN	CD (pediatric)	↑ *Oscillibacter*, *Roseburia*, *Firmicutes*, *Clostridiales*, *Clostridia*, *Ruminococcus*	↓ *Haemophilus*, *Veillonella*, *Anaerostipes*, *Prevotella*, *Gammaproteobacteria*, *Proteobacteria*, *Escherichia*, *Burkholderiales*, *Klebsiella*
Tasty and Healthy	CD (pediatric/young adult)	↑ *Faecalibacterium prausnitzii*, *Bacteroides uniformis*	↓ *Ruminococcus gnavus* (in EEN group, not in T&H)
Vegetarian Diet	UC	↑ *Blautia*, *Coprococcus*, *Dorea*, *Ruminococcus*	↓ *Faecalibacterium prausnitzii*
Gluten-Free Diet	CD and UC		↓ *Barnesiellaceae*, *Clostridiales*, *Ruminococcus*, *Faecalibacterium prausnitzii*, *Bacteroidetes genera* (in CD)
High-Fiber (oligofructose-inulin)	UC	↑ *Bifidobacteriaceae*, *Lachnospiraceae*	↓ *Bacteroidaceae (mucosal)*
Low-Sulfur Diet	UC	↑ *Collinsella stercoris*, *Asaccharobacter celatus*, *Alistipes finegoldii*, *Faecalibacterium prausnitzii*, *Bacteroides ovatus*, *Parabacteroides distasonis*, *Blautia wexlerae*, *Agathobaculum butyriciproducens*	↓ *Eggerthella lenta*

**Table 3:** Potential drug–nutrient interactions.

Drug Class	Affected Micronutrients	Supplementation Recommendations
Corticosteroids (systemic)	Calcium, Vitamin D, Potassium, Magnesium	Calcium 1000–1200 mg daily; Vitamin D 600–800 IU daily (higher doses may be needed to achieve 25-hydroxyvitamin D > 50 nmol/L or >30 ng/dL); Monitor and supplement potassium and magnesium as needed; Note: Budesonide and beclomethasone (10–15% systemic absorption) have substantially lower effects
Aminosalicylates (Sulfasalazine)	Folate	Folic acid 1 mg daily (standard); Serum folate monitored annually; Women of childbearing age: 2 mg daily for 3 months before conception (on sulfasalazine) or 5 mg daily (with extensive small bowel disease, malabsorption, or family history of neural tube defects)
Methotrexate	Folate (primary), Vitamin B12, Vitamin B6 (potential)	Folic acid 5 mg once weekly 24–72 h after methotrexate OR 1 mg daily for 5 days per week (excluding methotrexate day); Discontinue methotrexate 1–3 months before conception; Monitor micronutrients every 3–6 months
Thiopurines (Azathioprine, 6-mercaptopurine)	No true deficiency—causes macrocytosis through myelosuppression	No routine prophylactic supplementation beyond standard IBD screening; Maintain adequate iron, vitamin B12, folate, protein intake; Regular complete blood count monitoring; Can be continued during pregnancy
Biologic therapies (Anti-TNF, Vedolizumab, Anti-IL12/23, Anti-IL23)	No direct micronutrient depletions; Malnutrition and sarcopenia affect treatment outcomes	Address malnutrition and sarcopenia to improve treatment response; Nutritional support (adequate protein, albumin > 35 g/L) improves biologic efficacy; Consider combining biologics with nutritional interventions; Fiber intake supports beneficial microbiome
Small molecules (JAK inhibitors, S1P modulators)	No direct micronutrient depletions; JAK inhibitors may affect hematologic parameters and lipids	JAK inhibitors: Ensure adequate iron, folate, vitamin B12, protein status; Monitor lipids and consider heart-healthy diet; S1P modulators: No specific supplementation requirements based on current evidence

## Data Availability

Data sharing is not applicable.

## Abbreviations

The following abbreviations are used in this manuscript:

AhR	Aryl Hydrocarbon Receptor
BMI	Body Mass Index
CD	Crohn’s Disease
CDED	Crohn’s Disease Exclusion Diet
CurQD	Curcumin-QingDai
DEXA	Dual-Energy X-ray Absorptiometry
ECCO	European Crohn’s and Colitis Organisation
EDIP	Empiric Inflammatory Dietary Potential
EEN	Exclusive Enteral Nutrition
ESPEN	European Society for Clinical Nutrition and Metabolism
FC	Fecal Calprotectin
FMT	Fecal Microbiota Transplantation
FODMAP	Fermentable Oligosaccharides, Disaccharides, Monosaccharides And Polyols
GLP-1	Glucagon-Like Peptide 1
IBD	Inflammatory Bowel Disease
IBS	Irritable Bowel Syndrome
LF	Low-FODMAP
MD	Mediterranean Diet
NF-κB	Nuclear Factor-Kappa B
PEN	Partial Enteral Nutrition
RCT	Randomized Controlled Trial
SCCAI	Simple Clinical Colitis Activity Index
SCFAs	Short-Chain Fatty Acids
TNF-α	Tumor Necrosis Factor-Alpha
UC	Ulcerative Colitis
UPF	Ultraprocessed Food
